# Plus- and Minus-End Directed Microtubule Motors Bind Simultaneously to Herpes Simplex Virus Capsids Using Different Inner Tegument Structures

**DOI:** 10.1371/journal.ppat.1000991

**Published:** 2010-07-08

**Authors:** Kerstin Radtke, Daniela Kieneke, André Wolfstein, Kathrin Michael, Walter Steffen, Tim Scholz, Axel Karger, Beate Sodeik

**Affiliations:** 1 Institute of Virology, Hannover Medical School, Hannover, Germany; 2 Institute of Molecular Biology, Friedrich-Loeffler-Institute, Greifswald-Riems, Germany; 3 Institute of Molecular and Cell Physiology, Hannover Medical School, Hannover, Germany; University of North Carolina at Chapel Hill, United States of America

## Abstract

Many viruses depend on host microtubule motors to reach their destined intracellular location. Viral particles of neurotropic alphaherpesviruses such as herpes simplex virus 1 (HSV1) show bidirectional transport towards the cell center as well as the periphery, indicating that they utilize microtubule motors of opposing directionality. To understand the mechanisms of specific motor recruitment, it is necessary to characterize the molecular composition of such motile viral structures. We have generated HSV1 capsids with different surface features without impairing their overall architecture, and show that in a mammalian cell-free system the microtubule motors dynein and kinesin-1 and the dynein cofactor dynactin could interact directly with capsids independent of other host factors. The capsid composition and surface was analyzed with respect to 23 structural proteins that are potentially exposed to the cytosol during virus assembly or cell entry. Many of these proteins belong to the tegument, the hallmark of all herpesviruses located between the capsid and the viral envelope. Using immunoblots, quantitative mass spectrometry and quantitative immunoelectron microscopy, we show that capsids exposing inner tegument proteins such as pUS3, pUL36, pUL37, ICP0, pUL14, pUL16, and pUL21 recruited dynein, dynactin, kinesin-1 and kinesin-2. In contrast, neither untegumented capsids exposing VP5, VP26, pUL17 and pUL25 nor capsids covered by outer tegument proteins such as vhs, pUL11, ICP4, ICP34.5, VP11/12, VP13/14, VP16, VP22 or pUS11 bound microtubule motors. Our data suggest that HSV1 uses different structural features of the inner tegument to recruit dynein or kinesin-1. Individual capsids simultaneously accommodated motors of opposing directionality as well as several copies of the same motor. Thus, these associated motors either engage in a tug-of-war or their activities are coordinately regulated to achieve net transport either to the nucleus during cell entry or to cytoplasmic membranes for envelopment during assembly.

## Author Summary

Many viruses, particularly neurotropic alphaherpesviruses such as herpes simplex virus (HSV), require an intact microtubule network for efficient replication and pathogenesis. In living cells, host and viral cargo show rapid reversals in transport direction, suggesting that they can recruit motors of opposing directionality simultaneously. To elucidate the molecular mechanisms for specific motor-cargo recognition, it is necessary to characterize the surface of such cargos. We established a cell-free system that reconstitutes the binding of native, mammalian microtubule motors to intact tegumented HSV capsids. Our data suggest that the inbound motor dynein and the outbound motor kinesin-1 bind directly and independently of other host factors to the inner tegument that coats the capsids during cytosolic transport. Identifying viral receptors for the hosts' transport machinery will provide us on the one hand with new potential targets for antiviral therapy. On the other hand, such viral protein domains could be added to viral vectors or even to artificial nano carriers designed to deliver therapeutic genes or molecules to the nucleus or other subcellular destinations.

## Introduction

To reach their destined subcellular location, viruses utilize motor proteins that move unidirectional along microtubules (MT) or actin filaments [1], [2], [3], [4], [5]. Digital time-lapse microscopy experiments in living cells have provided fundamental insights into the dynamics of intracellular transport, and demonstrated that host and viral cargos show rapid reversals in transport direction. This raises the question how net transport towards a particular destination is achieved and regulated. The cargo surface could either engage exclusively inbound or outbound motors according to the hypothesis of “exclusive presence”, or the capsids could recruit motors of opposing directionality simultaneously [6], [7], [8], [9]. Two other scenarios could then enable net-transport despite the simultaneous presence of different motors. According to the “tug-of-war” hypothesis, each motor species moves the cargo towards its own direction, and the net course is determined by those motors exerting a stronger force than the opposition [10], [11], [12]. The hypothesis of “coordinated regulation” proposes that the activity of the bound motors is coordinately regulated so that they do not interfere with each other [13], [14], [15].

Viral structures engage motors at different stages of the viral life cycle [1], [2], [3], [4]. Intracellular long distance motility such as axonal transport requires a special subgroup of MT associated proteins (MAPs), the MT motors. Cytoplasmic dynein, often in concert with its cofactor dynactin, moves towards the minus-ends of MTs in the cell center, while most kinesins propel towards MT plus-ends pointing towards the plasma membrane and the presynaptic terminals. These MAPs are built from multiple subunits derived from several genes and alternative splice forms resulting in a molecular weight of 1.5 MD for dynein, 1.2 MD for dynactin, 340 to 380 kD for kinesin-1, and 260 to 280 kD for kinesin-2 [9], [16], [17], [18].

Viral interactions with the host transport machinery can be direct or indirect: viral proteins either bind to specific motor subunits, or interact with membrane-associated and cytosolic adaptors to link to such motors. Furthermore, viral particles are transported within membranes that in turn recruit motors [3], [5], [8]. However, the protein composition of motile viral particles is often unknown, and may even change as the incoming viral genome is uncoated for transcription and replication, or as progeny genomes are packaged into virions [3], [4], [5]. Several interactions of motor subunits with viral proteins have been identified, but could not be confirmed in the context of native viral particles and functional motor complexes due to the lack of appropriate biochemical assays [3], [8]. Host proteins often show altered affinities in isolation than after incorporation into protein complexes. Dynein subunits, for example change their conformation upon assembly into the motor, and thus their potential to interact with putative cargo structures [19]. Furthermore, viral proteins are often difficult to produce in soluble form since they have evolved to self-assemble into higher ordered viral structures [20]. Their structure may refold upon assembly into viral particles, and at present, it is unclear which domains they expose to the cytosol.

Thus, there is a gap between studying the interactions of individual host and viral proteins in isolation, and the analysis of intracellular dynamics of viral and subviral structures in living cells. One powerful approach for functional analysis is to reconstitute a biological process from its components [21]. Yet, few studies have characterized the association of viral particles with cytosolic factors. Towards this end, we have developed cell-free biochemical assays to analyze binding of viral capsids to MT motors, capsid transport along MTs, capsid docking to nuclear pore complexes, and viral genome uncoating [22], [23]. For such experiments, we use capsids from different stages of the life cycle of herpes simplex virus type 1 (HSV1). Viral capsids purified from HSV1 virions, but not nuclear capsids, that are isolated from infected cells, recruit dynein and dynactin from *Xenopus laevis* cytosol. Consistent with these data, the *in vitro* transport of HSV1 capsids along MTs requires ATP and cytosolic host factors [23].

HSV1 belongs to the alphaherpesviruses that establish lifelong latent infections in sensory neurons after productive replication in skin and mucosa, and that are actively transported within axons to establish latency, after reactivation and during spread in the central nervous system [2], [24], [25]. HSV1 is the causative agent of *herpes keratitis*, the leading cause of blindness by infection, of *herpes encephalitis*, and of *eczema herpeticum* in patients with atopic dermatitis [24]. The HSV1 virion is characterized by a non-centric localization of the icosahedral capsid with a diameter of 125 nm that contains the DNA genome. The capsid is covered by the tegument that on its distal pole has a thickness of up to 35 nm, and by a viral envelope [26]. Different HSV1 structures are transported within cells: unenveloped cytosolic capsids, capsids within virions enclosed by cellular membranes, and possibly also capsids attached to the cytosolic face of host membranes [2], [25], [27], [28], [29], [30]. Fluorescently labeled particles of both HSV1 and pseudorabies virus (PrV), an alphaherpesvirus of pig, show bidirectional transport towards the cell center as well as the cell periphery with occasional, rapid reversals in direction [29], [31], [32; Janus & Sodeik, in preparation].

After fusion of the HSV1 envelope with a host membrane, dynein transports incoming cytosolic capsids along MTs towards the nucleus [26], [33], [34], [35], [36]. Following nuclear egress, progeny cytosolic capsids acquire their envelope in the cytoplasm, and subsequently complete mature virions inside membranes are transported to the plasma membrane for exocytic egress [4], [30], [37], [38]. HSV1 requires an intact MT cytoskeleton for egress and spread, and axonal progeny particles co-localize with kinesin-1 [25], [29], [30], [39], [40]. However, the protein composition of inbound or outbound viral particles is not known. Cytosolic HSV1 capsids could potentially use as many as 33 different capsid or tegument proteins to recruit MT motors that we have classified into six groups (Table 1).

**Table 1:** HSV1 proteins potentially interacting with cytosolic host factors.

HSV1 - structural protein	Copies/Virion	MW [kDa]	Method of Analysis *
**Capsid protein**			
VP5 (UL19) - hexon+penton	955	149	IB, MS
VP26 (UL35) - hexon surface	900	12	K/O, IB, MS
VP19c (UL38) - triplex	320	50	IB, MS
VP23 (UL18) - triplex	640	34	MS
VP24 (pUL26-N-term)	150	27	MS
**Minor capsid-associated protein** - required for DNA packaging			
pUL6 - portal	12	74	IB
pUL17	40	75	IB, MS
pUL25	82	63	IB, MS, IEM
**Inner tegument protein**			
pUS3 - protein kinase	?	53	K/O, IB, IEM
pUL36 - deubiquitinase	110–150	336	IB, MS, IEM
pUL37 (GFPUL37)	30–40	121	IB, MS, IEM
**Tegument protein with enzymatic activity**			
pUL13 - protein kinase	?	57	-
pUL23 - thymidine kinase	?	41	-
pUL39 - ribonucleotide reductase subunit	?	124	-
pUL40 - ribonucleotide reductase subunit	?	38	-
vhs (virus host shut off protein, UL41)	?	55	IB
pUL50 - dUTPase	?	39	-
**Other tegument protein**			
ICP0 (RL2)	?	79	IB
ICP4 (RS1)	?	133	IB
ICP34.5 (RL1)	?	26	IB
pUL7	?	33	-
pUL11 - myristylated	?	11	IB
pUL14	20–40	24	IB
pUL16	?	40	IB, MS
pUL21	?	58	MS
pUL51	?	26	-
pUL55	?	21	-
pUS2 - prenylated	?	33	-
pUS10	?	34	-
**Major tegument protein**			
VP11/12 (UL46)	410–1700	78	K/O, MS
VP13/14 (UL47)	1800	74	IB, MS, IEM
VP16 (UL48; αTIF)	1000–2000	54	IB, MS, IEM
VP22 (UL49)	1000–1500	32	IB, MS, IEM
pUS11 – binds RNA & ribosomes	600–1000	18	K/O, IB

The list of HSV1 structural proteins was compiled using several sources [23], [24], [30], [37], [45], [64], [98], [99]: K/O, HSV1 deletion mutant; IB, immunoblot; MS, quantitative SILAC mass spectrometry; IEM, quantitative immunoelectron microscopy.

Here, we show that mammalian dynein, dynactin, kinesin-1 and kinesin-2 were recruited to HSV1 capsids, and that dynein, dynactin and kinesin-1 bound directly and independently of other host proteins. We analyzed the protein composition and surface of five different HSV1 capsid types with respect to 23 of the 33 structural HSV1 proteins potentially exposed to the cytosol. Our data indicate that mammalian MAPs bound preferentially to capsids that exposed inner tegument proteins such as pUS3, pUL36, pUL37, ICP0, pUL14, pUL16 or pUL21 (pUL**X**, protein encoded by gene UL**X** on the unique long region of the HSV1 genome; pUS**Y**, protein encoded by gene US**Y** on the unique short region). Neither untegumented nuclear capsids nor capsids covered by outer tegument proteins bound to MAPs. Our data suggest that dynein, dynactin and kinesin-1 use three different inner tegument structural features to bind HSV1 capsids, and that an individual capsid can simultaneously accommodate motors of opposing polarity as well as several copies of the same motor.

## Results

### Probing the interaction of HSV1 capsids with cytosolic host factors

**Figure 1:**
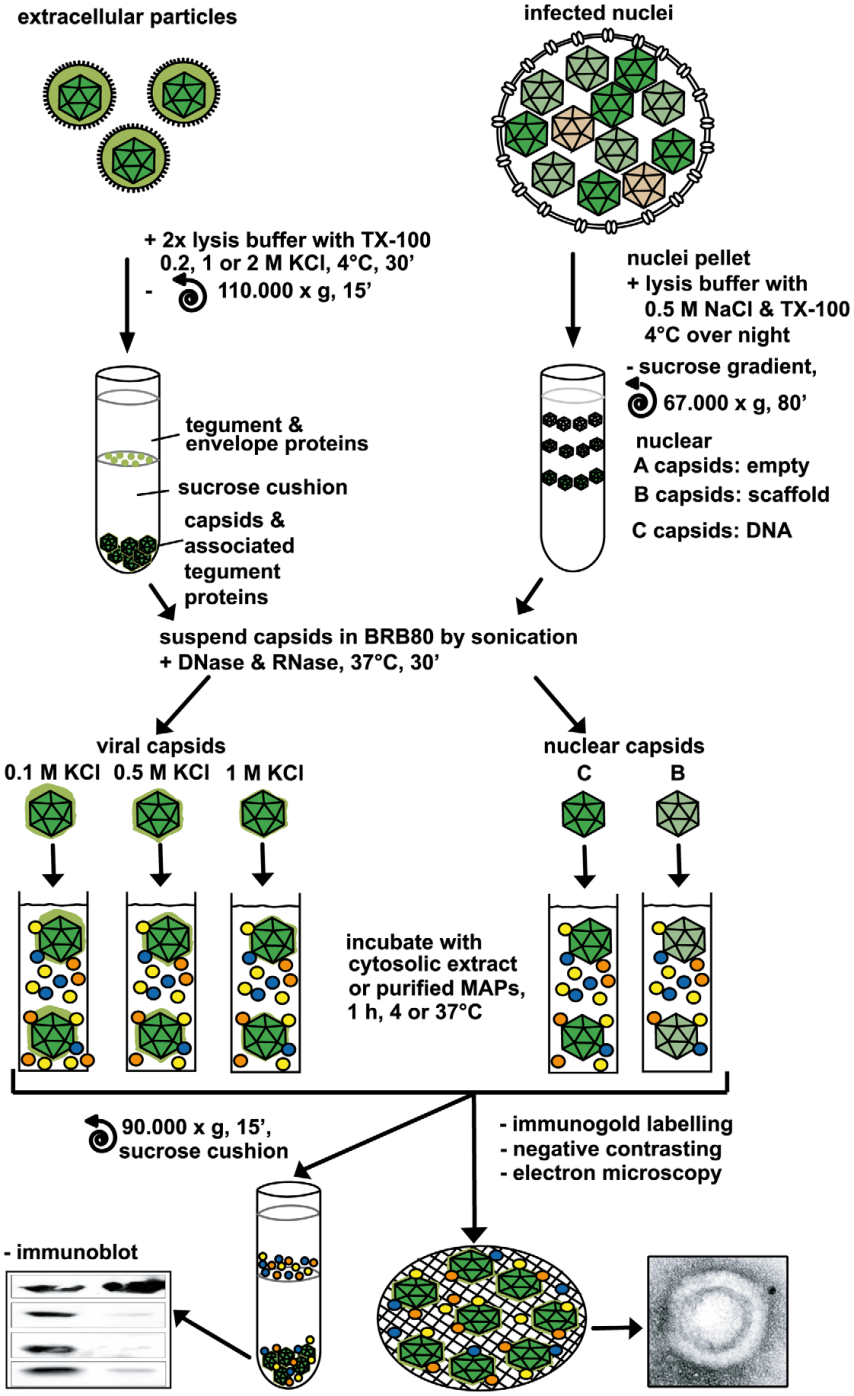
Analysis of host factor recruitment to HSV1 capsids. Partially tegumented viral capsids were generated from mature extracellular particles released from HSV1 infected cells by detergent lysis to remove the viral envelope in the presence of 0.1 M, 0.5 M or 1 M KCl to extract different amounts of tegument (light green), and purified through sucrose cushions. Untegumented nuclear B and C capsids (dark green) were isolated from the nuclei of HSV1 infected cells by gradient sedimentation. The 5 different capsid types were resuspended in BRB80 buffer using tip sonification, treated with DNase and RNase, repelleted, and then incubated with cytosolic extracts or purified MAPs (colored circles). Capsids were analyzed for bound host factors either after sedimentation through a sucrose cushion by immunoblot or directly by electron microscopy after immunolabeling and negative contrasting.

To generate HSV1 particles that potentially bind host factors, we extracted viral capsids with the non-ionic detergent TX-100 at three different KCl concentrations (0.1, 0.5 or 1 M) from extracellular particles, or isolated two different nuclear capsids (B or C capsids) from infected cells (Fig. 1). The supernatant of infected cells contains not only intact virions, but also unenveloped capsids, membrane vesicles and exosomes as well as enveloped L (light) particles containing HSV1 envelope and tegument but no capsid proteins [41], [42], [43], [44]. The viral particles sedimented from the medium of infected cells were treated with trypsin to remove potentially associated host factors, and subsequently with trypsin inhibitor to inactivate the protease prior to the lysis of the virions with detergent (c.f. Materials & Methods; Fig. S1).

As a source of mammalian proteins, we used cytosolic extracts of pig brain that contain large amounts of MT motors. HSV1 capsids were incubated with this cytosol, the mixtures were layered on sucrose cushions, and capsids with associated host proteins were sedimented. Extracellular viral particles equivalent to about 5×10^8^ plaque-forming units (PFU) and treated with trypsin were required to detect host proteins recruited from the cytosolic extract. Since each capsid contains a high copy number of viral proteins (c.f. Table 1), but bound only few MAPs, the signals for host proteins were lower than that for viral proteins which served as loading controls. As in the *Xenopus* system [23], mammalian dynein and dynactin were co-sedimented by HSV1 viral capsids from a cytosolic extract.

### Distinct HSV1 capsids associate with specific MAPs

**Figure 2:**
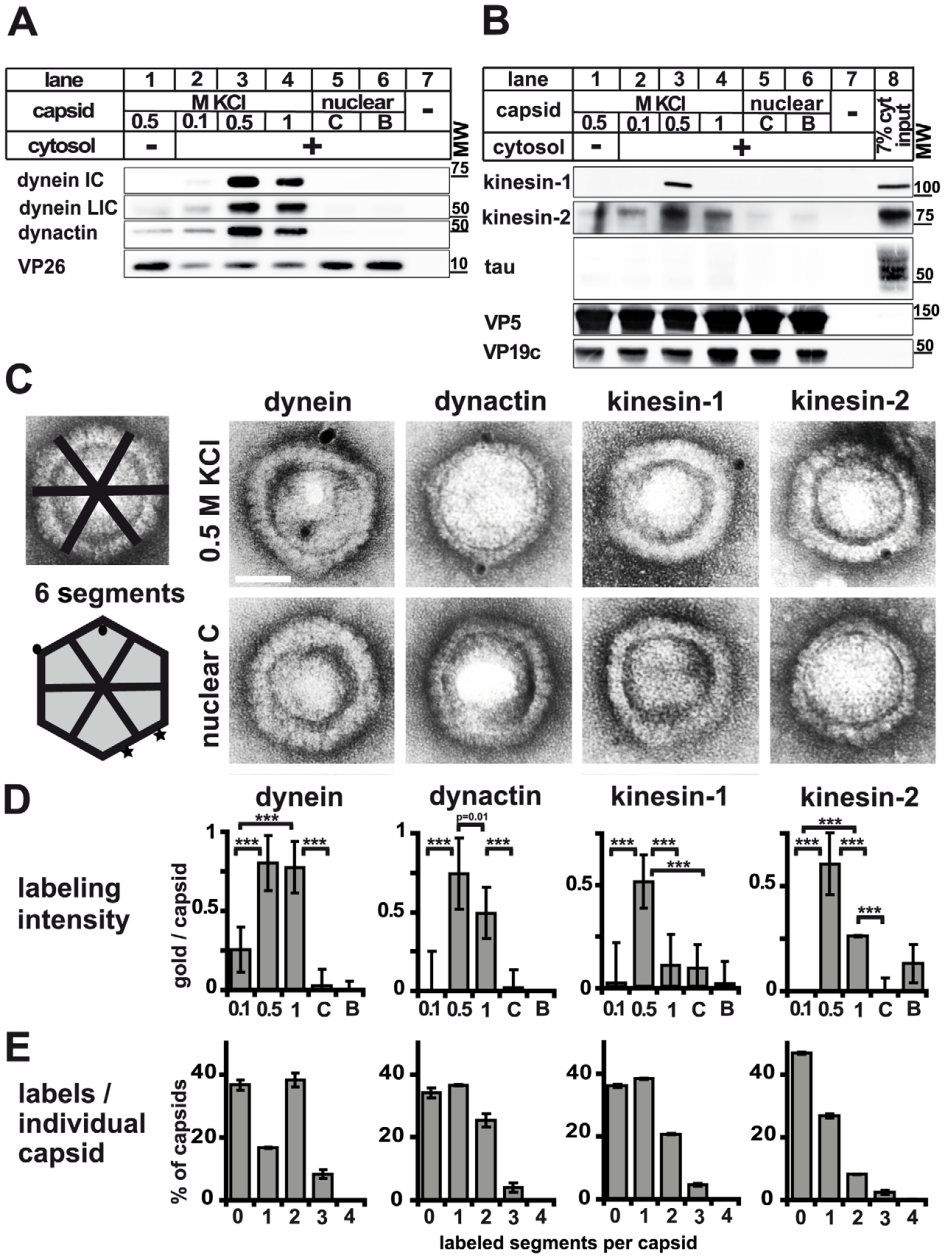
Plus- and minus-end directed MT motors bind to HSV1 capsids. **A** and **B**: Immunoblot analysis of MAP binding to capsids. HSV1(F) viral (0.5 M KCl, lanes 1 and 3; 0.1 M KCl, lanes 2; 1 M KCl, lanes 4) or nuclear capsids (C capsids, lanes 5; B capsids, lanes 6) or mock samples lacking capsids (lanes 7) were incubated in 0.25 mg/ml (A, lanes 2 to 7) or 0.75 (B; lanes 2 to 7) mg/ml pig brain cytosol, and sedimented through sucrose cushions. The host proteins were analyzed by immunoblot using antibodies against dynein (A: MAB1618 against intermediate chain; pAb anti-LIC2 against light intermediate chain), and dynactin (A: mAb anti-p50), or kinesin-1 (B: MAB1613 against heavy chain), kinesin-2 (B: mAb against KAP3A) and tau (B: pAb). The amount of capsids in each sample was estimated by labeling the major capsid proteins VP26 (A: pAb anti-VP26), VP5 (B: pAb NC-1), or VP19c (B: pAb NC-2). As a loading control, 7% of the amount of the input cytosol was also directly analyzed (B; lane 8). **C**: Immunoelectron microscopy images of HSV1(F) capsids incubated with 0.75 mg/ml pig brain cytosol and labeled with mouse monoclonal antibodies against dynein (MAB1618 against intermediate chain), dynactin (mAb anti-p50), kinesin-1 (MAB1613 against heavy chain) or kinesin-2 (mAb K2.4 against KIF3A) followed by rabbit-anti-mouse antibodies, protein-A gold and negative staining. Scale bar: 50 nm. **D**: Quantification of the labeling intensity of different capsid types. After immunogold-labeling with antibodies against MAPs, negative staining and electron microscopy, the number of gold particles per capsid was counted for 100 to 170 capsids. Labeling of capsids incubated with buffer instead of cytosol was considered background and subtracted. Error bars: SEM. Three asterisks denote P<0.0001 as determined in a two-sided Student's t-test. **E**: Labeling frequency for different MAPs on individual viral capsid treated with 0.5 M KCl. In a two-dimensional projection, the icosahedral capsid appears as a hexagon that can be divided into 6 segments (c.f. C; schematic capsid). The number of capsids that had labeling on 0, 1, 2, or more of such segments was counted. Gold particles were counted as multiple labeling events when they were more than 40 nm apart (C; circles on schematic capsid). Labels in closer proximity were scored as only one label (C; stars on schematic capsid). Error bars: SEM. The quantifications (D, E) were compiled from three experiments analyzing dynein, dynactin and kinesin-1 and two experiments for kinesin-2.

We then addressed which HSV1 capsids bind mammalian MT motors. While nuclear B or C capsids do not contain tegument proteins, viral capsids isolated by TX-100 lysis from extracellular virions contain significant amounts of tegument [23], [45]. With the salt concentration increasing from 0.1 M KCl to 0.5 or even 1 M, several major tegument proteins detach from the viral particles, while inner tegument proteins continue to remain associated [23]. All 5 capsid types contained the major capsid proteins VP5, VP19c and VP26 (Fig. 2A,B, VP: viral protein). The viral capsids (Fig. 2C) had a morphology similar to nuclear C capsids as seen in electron microscopy [46], suggesting that the tegument did not adsorb further uranyl acetate that was used for negative contrasting.

We next analyzed the different capsids for their ability to interact with mammalian factors. The MAPs dynein (Fig. 2A), dynactin (Fig. 2A), kinesin-1 (Fig. 2B) and kinesin-2 (Fig. 2B) bound to HSV1 viral capsids treated with 0.5 M KCl. While kinesin-1 exhibited a remarkable binding preference for 0.5 M KCl capsids, dynein, dynactin and kinesin-2 also showed a strong affinity for capsids isolated at 1 M KCl. In contrast, tau, a non-motor MAP, did not associate with any capsid type (Fig. 2B). Nuclear B or C capsids neither bound dynein, dynactin, kinesin-1, nor kinesin-2. Viral capsids extracted at 0.1 M KCl also recruited only a few host factors. Moreover, recombinant GST or other cytosolic proteins such as tau, amphiphysin-1, and LC3 also did not bind to any of the capsids (not shown). Thus, some but not all MAPs bound to a specific subset, but not to all HSV1 capsids.

Although we re-suspended the capsids by repeated tip sonification and DNase and RNase treatment (Fig. 1), they may oligomerize to different degrees that would reduce the accessible capsid surface area. Therefore, we performed single capsid analysis using electron microscopy. Nuclear capsids had a lower propensity than viral capsids to associate with each other, suggesting that the presence of tegument increased the affinity of capsids for each other. All viral capsid preparations contained many mono-dispersed capsids, but also some larger capsid assemblies. However, the ratio of monomers to oligomers and aggregates was independent of the applied KCl concentration (data not shown). Since there was little oligomerization of nuclear capsids, and that of viral capsids was independent of the tegument composition, differences in MAP binding between different capsid types were not caused by differences in capsid aggregation.

To study motor binding to individual particles, we adsorbed capsids suspended in cytosol onto electron microscopy grids and labeled them with antibodies against MAPs that in turn were detected by protein A-conjugated colloidal gold (Fig. 1). Gold particles co-localizing with mono-disperse capsids were counted to quantify the amount of host factors per capsid (Fig. 2C). To exclude an overestimation based on aggregated gold associated with only one antigen or on multiple gold labels on the multiple subunits of a single motor complex [33], [47] multiple gold particles less than 40 nm apart were only scored as one label (Fig. 2C; circles on the capsid drawing represented two labeling events, whereas the stars were recorded as one label). The average number of gold particles on capsids that had been incubated with buffer instead of cytosolic proteins was considered background and subtracted. Capsids treated with 0.5 M KCl had 0.75 gold/capsid above background after labeling for dynein and dynactin, and 0.5 gold/capsid for kinesin-1 and kinesin-2 (Fig. 2D). Consistent with the immunoblot data, the affinity of dynein for 0.5 M KCl capsids was as strong as for 1 M KCl capsids, whereas 0.1 M KCl and nuclear capsids recruited little dynein. Binding of dynactin and kinesin-2 was similar to that of dynein, but there was less binding to 1 M than to 0.5 M KCl capsids. In contrast, kinesin-1 was most prominently detected on capsids extracted at 0.5 M KCl, whereas all other capsid types had recruited very little kinesin-1.

**Figure 3:**
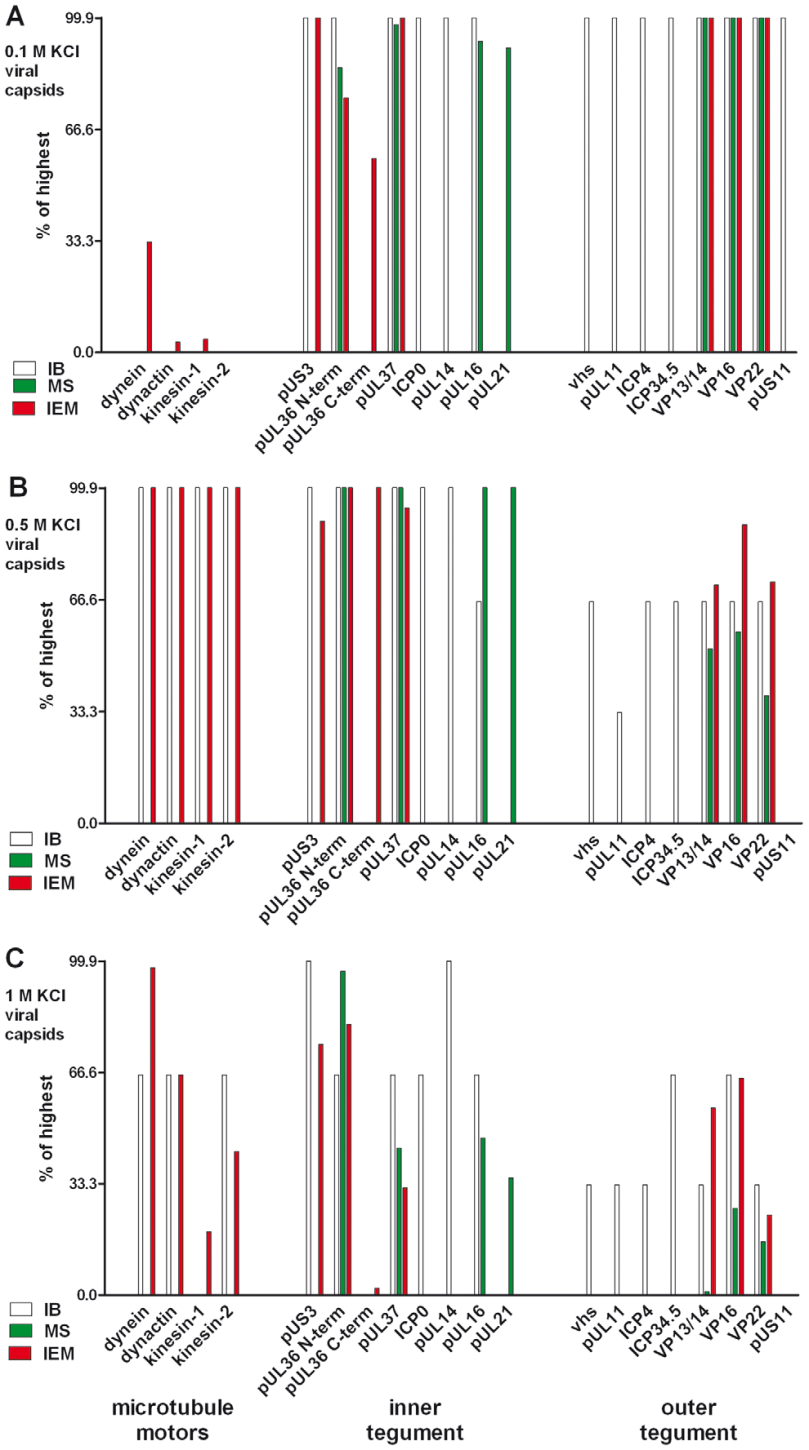
Characterization of tegumented, viral HSV1 capsids. The MAP binding (Fig. 2; left 4 data sets) to the three viral capsid types treated with 0.1, 0.5 or 1 M KCl (panels **A**, **B** and **C**, respectively) and their inner (middle 8 data sets) and outer tegument (right 8 data sets) organization (Figs. 6,7,8) have been analyzed by immunoblot (IB), quantitative mass spectrometry (MS), and quantitative immunoelectron microscopy (IEM). While IB and MS indicate the amount of different tegument proteins on the capsids, the IEM determines to what extent such tegument proteins were accessible on the capsid surfaces to antibodies or host factors. Please note that nuclear capsids did not bind to MAPs and contained very little tegument (c.f. Figs. 2, 6, 7, 8; data not shown in this figure). For further comparative analysis, we normalized the results of the IB, MS and IEM for the viral capsids such that the amount of a given MAP or a tegument protein on the capsids with the highest amount was set to 100%, and recalculated accordingly for the other capsid types (% of highest). These results are also listed in supplementary Table S1. Please note that in contrast to MS and IEM, the IB data were not quantitative. Instead, we only estimated the amount of the respective proteins based on the band intensities into 4 classes: absent (0%), minor (33%), major (66%) or highest (100%) amounts.

For further assessment, we normalized the results of the immunoblot and the immunoelectron microscopy for the viral capsids such that the amount of a given MAP on the capsids with the highest amount was set to 100%, and the other data recalculated accordingly (Fig. 3, left 4 data sets; Supplementary Table S1). The quantitative single-particle analysis confirmed that the minor differences in capsid aggregation did not account for the specificity of MAP binding to HSV1 capsids. All MAPs preferentially bound to capsids that had been extracted with high KCl concentrations, but not to nuclear, un-tegumented capsids, or to 0.1 M KCl capsids containing the highest amount of tegument proteins. Overall, the viral capsids treated with 0.5 M KCl treatment provided the best substrate for MAP binding.

### Several motors can bind to one HSV1 capsid

The electron microscopy analysis prompted us to ask whether a viral capsid treated with 0.5 M KCl could bind several copies of a MAP simultaneously. In a two-dimensional projection, icosahedral capsids appear as hexagons that we divided into six segments (Fig. 2C, capsid drawing). We then counted the number of capsids that had labels on 0, 1, 2, or more of such segments as a means for the potential presence of several host factors per capsid. We never detected labeling on 5 or all 6 segments (Fig. 2E). There were two labels for dynein on about 40% of the capsids, for dynactin on 25%, for kinesin-1 on 20% and for kinesin-2 on 8%. There were also a few capsids with even three labels for dynein, dynactin, or kinesin-1. Taking the background labeling into account (about one gold on every second or third capsid), these data suggest that we could detect at least more than one complex of dynein on about 20%, and more than one dynactin and kinesin-1 on about 10% of the capsids.

### Dynein, dynactin, and kinesin-1 bind directly to HSV1 capsids

**Figure 4:**
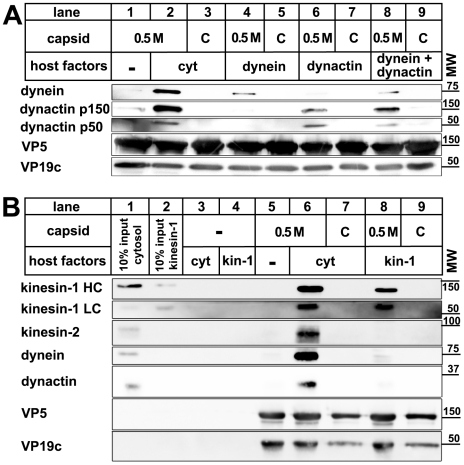
Dynein, dynactin, and kinesin-1 bind directly to tegumented HSV1 capsids. HSV1 (F) capsids binding do dynein and or dynactin (**A**) or kinesin-1 (**B**) was analyzed as follows: HSV1(F) capsids generated by detergent lysis of extracellular virions in the presence of 0.5 M KCl, or nuclear C capsids were incubated with buffer (A: lane 1), 0.25 mg/ml pig brain cytosol (A: lanes 2 and 3), 15 µg/ml purified, native dynein (A: lanes 4 and 5), 15 µg/ml purified, native dynactin (A: lanes 6 and 7) or native dynein and dynactin (A: lanes 8 and 9) or with buffer (B: lane 5), 0.75 mg/ml pig brain cytosol (B: lanes 6 and 7) or 33 µg/ml purified, native kinesin-1 (B: lanes 8 and 9), and sedimented through a sucrose cushion. As controls, cytosol (B: lanes 1 and 3) or purified kinesin-1 (B: lanes 2 and 4) were also directly analyzed (B: lanes 1 and 2) or sedimented in the absence of capsids (B: lanes 3 and 4). Host proteins were detected by immunoblotting with antibodies against dynein (A & B: MAB1618 against intermediate chain), dynactin (A: mAb anti-p150, mAb anti-p50, B: anti-CapZβ mAb3F2.3), kinesin-1 (B: MAB1613 against heavy chain, MAB1616 against light chain), kinesin-2 (B: mAb against KAP3A). As loading controls, the samples were probed with antibodies against the capsid proteins VP5 (A and B: pAb NC-1) or VP19c (A and B: pAb NC-2). These blots show one of three independent experiments yielding similar results.

Transport of cellular cargo often requires adaptors to link the cargo to its motor [6], [9], [17]. To test whether MAP binding required additional factors, we incubated capsids with purified motor complexes. Native dynein, dynactin, or kinesin-1 bound directly to 0.5 M KCl viral capsids, but not to nuclear capsids (Fig. 4). When we applied dynein and dynactin together, there was neither a synergistic increase nor a reduction in binding of these MAPs to the viral capsids. Thus, dynactin was not required to recruit dynein to HSV1 capsids. In addition, dynein and dynactin did not seem to compete for the same capsid binding sites. These data show that no further host adaptors were required for recruiting dynein, dynactin, or kinesin-1 to HSV1 viral capsids.

### Motor binding does not require the HSV1 proteins VP26, pUS11, or VP11/12

**Figure 5:**
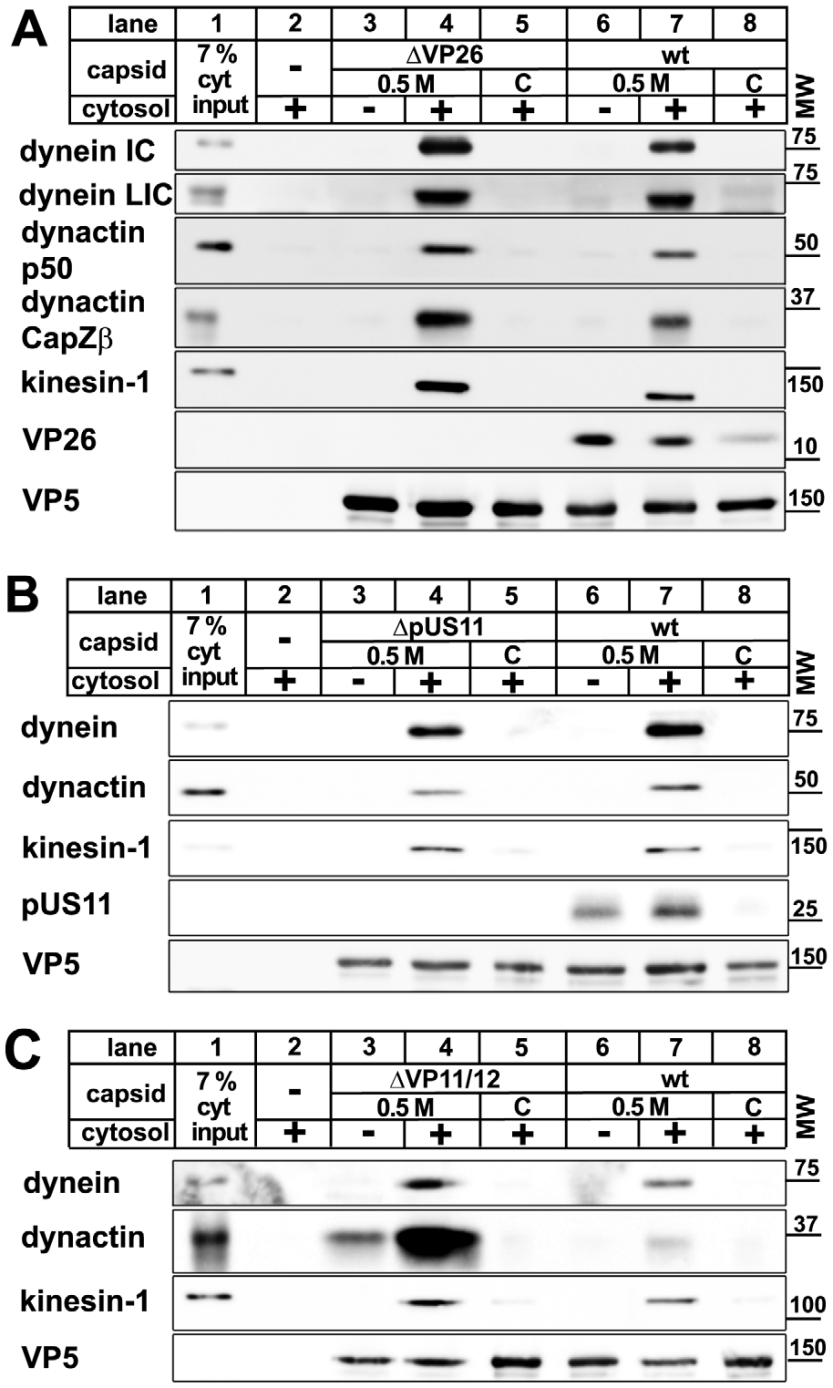
MAPs bind to HSV1 capsids lacking VP26, pUS11, or VP11/12. Capsids of HSV1(KOS)-ΔVP26 (**A**: lanes 3 to 5), HSV1(F)-ΔUS11 (**B**: lanes 3 to 5), or HSV1(F)-ΔVP11/12 (**C**: lanes 3 to 5) were isolated by detergent lysis of trypsin-treated extracellular virions in the presence of 0.5 M KCl or from nuclei of infected cells (C capsids). HSV1(F) wild-type was used as control (A, B, C: wt, lanes 6 to 8). The capsids were incubated in 0.75 mg/ml pig brain cytosol, and after sedimentation analyzed by immunoblot with antibodies against dynein (A, B, C: MAB1618 against intermediate chain; A: pAb anti-LIC2 against light intermediate chain), dynactin (A, B: mAb anti-p50; A, C: mAb3F2.3 against CapZβ), or kinesin-1 (A, B, C: MAB1613 against heavy chain). Labeling with antibodies against VP26 (A: pAb anti-VP26) or pUS11 (B: mAb #28) confirmed the lack of these proteins on mutant capsids. As loading control, the samples were probed with antibodies against the capsid protein VP5 (A, B, C: pAB NC-1). As controls, cytosol alone was directly analyzed (A, B, C: lanes 1) or sedimented in the absence of capsids (A, B, C: lanes 2). These blots show one of two or more experiments yielding similar results.

Several HSV1 proteins have been implicated in intracellular transport. The major capsid protein VP26 can directly interact with dynein light chains [48], and the major tegument protein pUS11 with kinesin-1 heavy chain (KIF5B) as well as the MT-binding protein PAT1 [49], [50]. We therefore generated capsids from HSV1 mutants lacking VP26, pUS11, or VP11/12 as another major tegument protein, and tested them for their ability to interact with MAPs. Dynein, dynactin and kinesin-1 bound viral capsids lacking VP26, pUS11, or VP11/12 but not the corresponding nuclear capsids (Fig. 5) indicating that the preference of MAP binding to tegumented capsids over untegumented ones was not due to these HSV proteins. Interestingly, in the absence of VP11/12, the MAPs bound with even higher efficiency. Thus, abundant tegument proteins such as VP11/12, or other proteins that require VP11/12 for binding to capsids, might prevent or reduce access to potential MAP receptors on the surface of the HSV1 capsids.

### Protein composition of nuclear and viral HSV1 capsids

**Figure 6:**
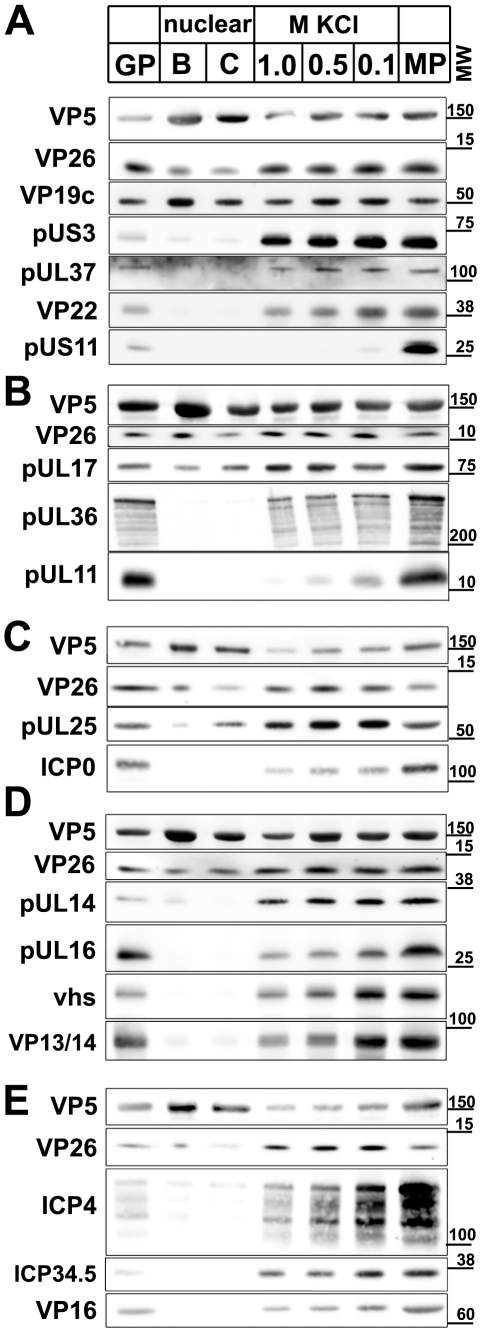
Immunoblot characterization of nuclear and viral HSV1 capsids. The protein composition of HSV1 capsids (nuclear B capsids, nuclear C capsids, viral capsids treated with 1.0, 0.5 or 0.1 M KCl), virions sedimented from cell culture supernatants (MP, medium pellet) and gradient purified virions (GP) were analyzed by immunoblot using antibodies directed against the capsid proteins VP5 (A–E: pAb NC-1), VP26 (A–E: pAb anti-VP26), VP19c (A: pAb NC-2), pUL17 (B: mAb #203), pUL25 (C: pAb ID1), pUL16 (D: anti-pUL16), or the tegument proteins pUS3 (A: pAb anti-pUS3), pUL37 (A: pAb 780), VP22 (A: pAb AGV30), pUS11 (A: mAb #28), pUL36 (B: pAb #147; anti-middle-pUL36), pUL11 (B: pAb anti-pUL11), ICPO (C: mAb 11060), pUL14 (D: anti-pUL14), vhs (D: pAb 11.388), VP13/14 (D: pAb R220), ICP4 (E: mAb 58S), ICP34.5 (E: pAb anti-ICP34.5) and VP16 (E: pAb SW7). Each blot shows one of three indendent experiments. The panels **A**, **B**, **C**, **D**, **and E** represent separate membranes. These data were after a further normalization also integrated into Fig. 3 (white columns).

**Figure 7:**
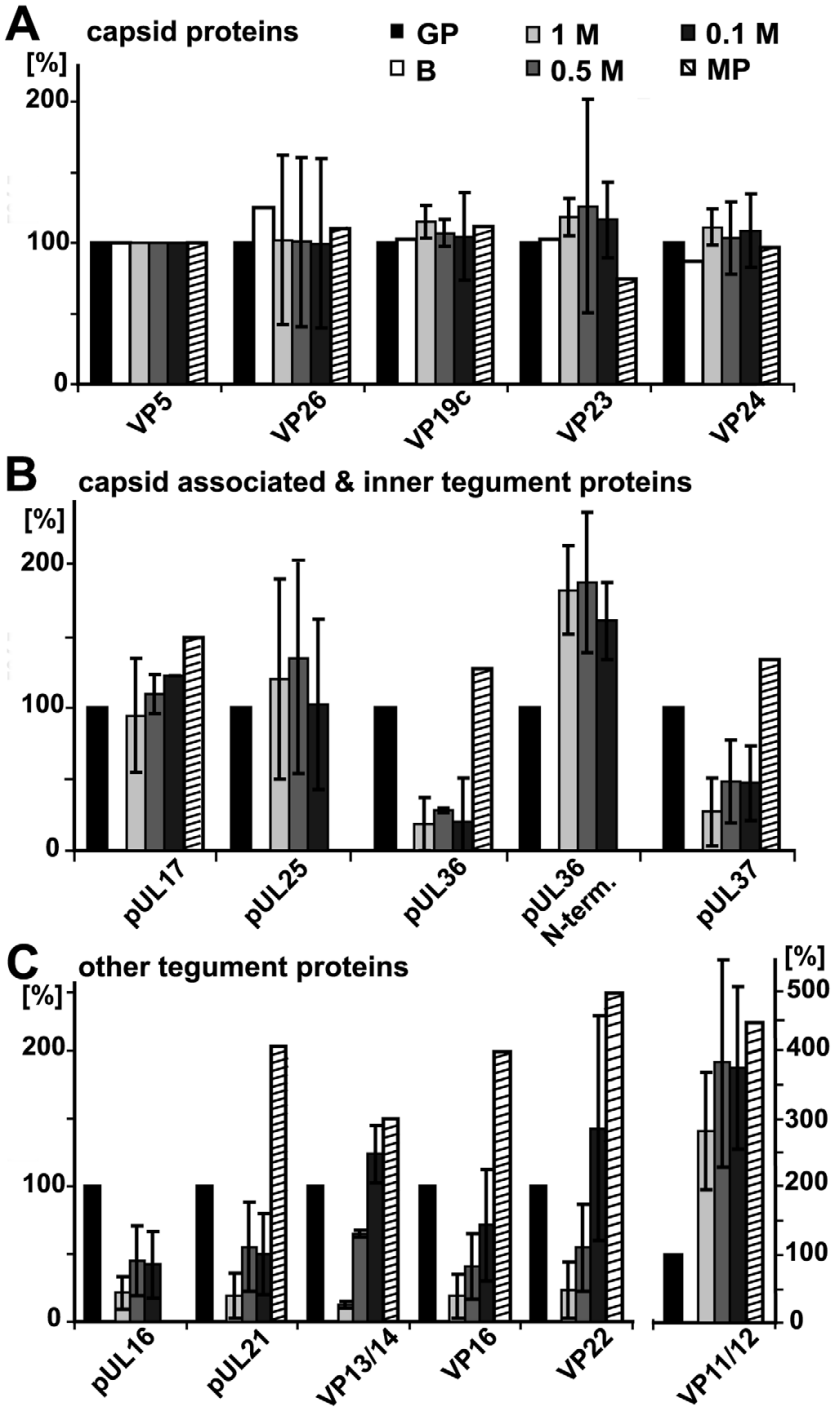
Mass spectrometric characterization of nuclear and viral HSV1 capsids. The protein composition of HSV1 capsids (B: nuclear B capsids, viral capsids treated with 1.0, 0.5 or 0.1 M KCl), virions sedimented from cell culture supernatants (MP, medium pellet) and gradient purified virions (GP) were analyzed by quantitative mass spectrometry. **A**: the capsid proteins VP5, VP26, VP19c, VP23, VP24, **B**: the capsid-associated proteins pUL17 and pUL25 and the inner tegument proteins pUL36, an N-terminal fragment of pUL36, pUL37, pUL16, pUL21 and **C**: the other tegument proteins pUL16, pUL21, VP13/14, VP16, VP22 and VP11/12 were analyzed. The relative protein amount in a capsid or a virus preparation is given in comparison to the gradient purified virions set as 100%. Values lower than 100% indicate removal of a protein in the respective capsid or virus preparation, whereas values higher than 100% indicate a higher amount of this protein in capsid samples than in gradient purified virions. Values are normalized to the amount of the capsid protein VP5 to account for varying amounts of capsids in each sample. Mean values of three independent experiments are given for viral capsids treated with 1, 0.5 and 0.1 M KCl, and one experiment for medium pellet and nuclear B capsids. Error bars: SD. These data were after a further normalization also integrated into Fig. 3 (green columns).

Next, we characterized the protein composition of the capsids. Our previous study has shown that major tegument proteins remain attached to viral capsids prepared at 0.1 M KCl, but are extracted with 0.5 M KCl; however, back then we detected no differences between 0.5 and 1 M KCl capsids [23]. Yet, 0.5 M KCl capsids bound kinesin-1 whereas those treated with 1 M did not. We therefore extended our initial immunoblot analysis, and performed quantitative mass spectrometry using stable isotope labeling with amino acids in cell culture (SILAC) [51] of (i) virions purified on linear nycodenz gradients (GP in Fig. 6 and 7), (ii) nuclear B capsids, (iii) nuclear C capsids, (iv) 1 M KCl viral capsids, (v), 0.5 M KCl viral capsids, (vi) 0.1 M KCl capsids, and (vi) trypsin-treated extracellular particles (MP for medium pellet), the starting material for preparation of viral capsids. We could thus analyze 23 of the 33 HSV1 proteins potentially exposed to cytosolic host proteins.

In addition to the specific proteins of interest, all blots were probed for the capsid proteins VP5 and VP26 as loading controls (Fig. 6). For mass spectrometry, each sample was mixed with deuterated gradient purified virions (GP in Fig. 7), and the relative concentration of proteins were determined from the ratio of deuterated (set to 100%) to undeuterated peptide peaks. If a capsid population contained less of a given protein than gradient purified virions, its value was below 100%. For standardization, the amount of VP5 in all capsid types was normalized to 100% [52], and the concentrations of the other HSV1 proteins were re-calculated accordingly (Fig. 7). When compared to the loading control VP5, all capsid types contained similar amounts of the capsid proteins VP19c, VP23, and VP24. While mass spectrometry indicated similar amounts of VP26 (Fig. 7A), the immunoblots showed a lower signal on nuclear than on viral capsids possibly suggesting an antibody preference for a mature form of VP26 (Fig. 6A–E). As reported before [53], [54], there were less pUL17 and pUL25 on B than on other capsid types (Fig. 6B,C).

The inner tegument proteins pUS3, pUL36, and pUL37 were present in similar amounts on viral, but absent from nuclear capsids. Compared to gradient purified virions, viral capsids contained less pUL36 and pUL37 (Fig. 6A,B; 7B), and interestingly, during capsid preparation, the amount of a 220 kD N-terminal fragment of pUL36 (residues 1 to about 2500; Fig. 6B) increased as full-length pUL36 decreased. A similar pUL36 fragment has also been detected in PrV virions [55]. An antibody directed against a middle portion of pUL36 (residues 1408–2112) detected numerous fragments of pUL36 (Fig. 6B), and Coomassie gels revealed two prominent bands of 270 and 220 kD (not shown). In addition, the HSV1 proteins ICP0, pUL14, pUL16, and pUL21 were present on viral capsids in similar amounts, with the lowest concentration on 1 M KCl capsids, but absent from nuclear capsids (Fig. 6C,D; 7C).

Nuclear capsids also lacked the major tegument proteins VP13/14, VP16, VP22, pUS11 and pUL11, but in contrast to inner tegument proteins, the concentration of these proteins was lowered with increasing KCl concentrations from 0.1 to 1 M KCl (Fig. 6A,B,D,E). Surprisingly, there was more VP11/12 in extracellular particles and viral capsids than in gradient-purified virions (Fig. 7C). The medium pellet also contained a higher proportion of pUL17, pUL36, pUL37, pUL21, VP13/14, VP16 and VP22 than gradient purified virions. This large amount of tegument proteins may originate from extracellular L particles that have a similar morphology as virions but lack capsids [41]. Tegument proteins derived from L particles, particularly VP11/12, might have associated with capsids during the lysis of the extracellular particles. vhs, ICP4, and ICP34.5 were also - in addition to VP13/14, VP16, VP22, pUL11, and VP11/12 - gradually detached from the viral capsids with increasing KCl amounts, but not detected on nuclear capsids (Fig. 6C,D,E).

For further assessment, we set the amount of a given tegument protein on the capsids with the highest amount to 100%, and recalculated the other data accordingly (Fig. 3; immunoblot, white columns; mass spectrometry, green columns; Supplementary Table S1). In summary, nuclear capsids that do not bind MAPs contained very little tegument. With salt concentrations increasing from 0.1 to 0.5 M KCl, major and many other tegument proteins were removed from the viral capsids, and the capsids bound better to MAPs. All viral capsids contained the inner tegument proteins pUS3, pUL36, an N-terminal fragment of pUL36, and pUL37, as well as ICP0, pUL14, pUL16, and pUL21 in similar amounts, albeit their amount was lower on 1 M than on 0.5 M KCl capsids.

### HSV1 capsids with different surface features

**Figure 8:**
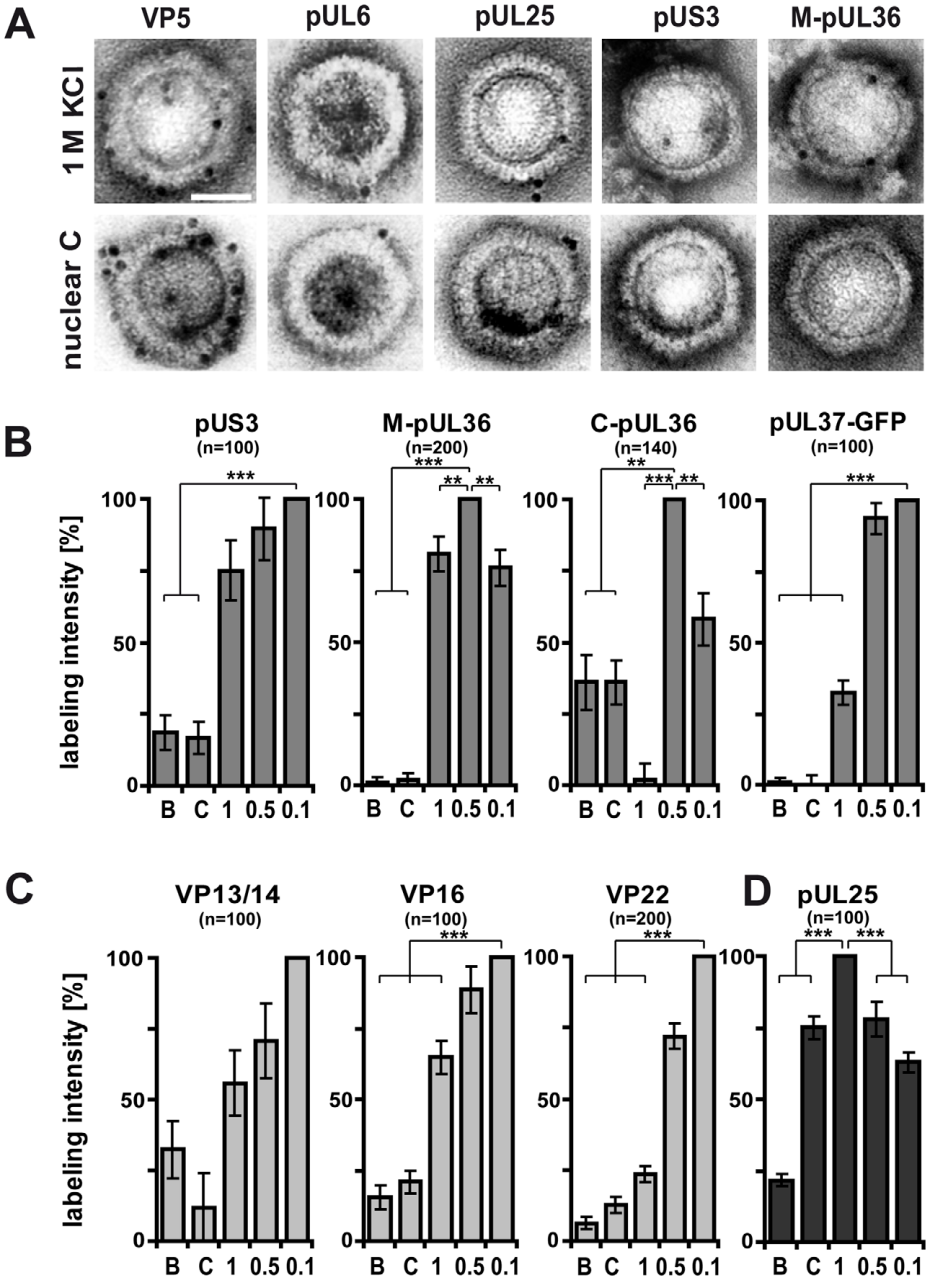
Generation of different HSV1 capsid surfaces during isolation. HSV1(F) capsids were isolated from infected nuclei (B or C) or prepared from extracellular virions by detergent lysis in the presence of different KCL concentrations (1, 0.5 or 0.1 M KCl), and labeled with antibodies against the capsid proteins VP5 (A: pAb NC-1), pUL25 (A, D: pAb ID1) or pUL6 (A: mAb 1C), against the inner tegument proteins pUS3, (A: pAb anti-US3), pUL36 (A, B: pAb #147, anti-middle-pUL36; B: pAb anti-Cterminal-pUL36), pUL37-GFP (B: mAb anti-GFP JL-8; here capsids from strain HSV1-pUL37GFP), or the outer tegument proteins VP13/14 (C: pAb R220), VP16 (D: pAb SW7), or VP22 (D: pAb AGV30). **A**: Immunoelectron microscopy images of capsids after immunolabeling followed by protein-A gold and negative staining. Scale bar: 50 nm. **B to D**: The labeling intensity for inner tegument proteins (B), outer tegument proteins (C) or the capsid associated protein pUL25 (D) was quantified by counting the number of gold particles per capsid. After subtraction of the background without the primary antibodies, the number on the capsid type with the highest labeling was set to 100%, and recalculated accordingly for the other capsid types. These data sets were also directly integrated into Fig. 3 (red columns). Error bars: SEM. n: number of capsids (same for each capsid type with a given antibody) summarized from three experiments. Two asterisks denote p<0.001 and three asterisks indicate P<0.0001 as determined in two-sided Student's t-tests.

The immunoblot and mass spectrometry analysis demonstrated that increasing KCl from 0.1 to 0.5 M extracted several tegument proteins from viral capsids, and thus presumably improved the accessibility of inner tegument structures (Fig. 3 and Supplementary Table S1). Capsids treated with 0.5 M or 1 M KCl had qualitatively similar, although quantitatively distinct, protein compositions, but differed substantially in recruiting kinesin-1. We wondered whether increasing to 1 M KCl might denature epitopes of the inner tegument without removing further proteins, and therefore examined the capsid surfaces by immunoelectron microscopy using various anti-HSV1 antibodies (Fig. 8A). All capsids were prominently labeled by antibodies directed against the major capsid protein VP5, whereas pUL6, the portal for DNA packaging [56] was detected at just one corner of the hexagonal capsid. For further assessment, we set after background subtraction the accessibility of given tegument epitopes on the capsids with the highest amount to 100%, and recalculated the other data accordingly (Fig. 8B–D; Fig. 3, red columns; Supplementary Table S1). Due to lack of suitable antibodies to pUL37, we also used HSV1 with a green fluorescent protein (GFP)-tagged pUL37 and an antibody directed against GFP.

The inner tegument proteins pUS3, pUL36 or pUL37GFP were not restricted to vertices. pUS3 was detected on all capsid types, but was much more abundant on viral than on nuclear capsids (Fig. 8B). While immunoblot and mass spectrometry analysis (Fig. 6B & 7B) showed similar amounts of pUL36 and the N-terminal pUL36 fragment on viral capsids, the immunoelectron microscopy revealed differences in pUL36 surface exposure. An antibody raised against a middle portion of pUL36 (residues 1408–2112) that detects numerous forms of pUL36 by immunoblot (Fig. 6B), showed a similar surface exposure of pUL36 (M-pUL36; Fig. 8A,B) on viral capsids with the highest signal on 0.5 M KCl capsids. In contrast, an antibody generated against a C-terminal peptide of pUL36 (residues 3048–3057; [57]) revealed the strongest signal on 0.5 M KCl capsids and an intermediate one on 0.1 M KCl capsids, but did not label 1 M KCl capsids at all (Fig. 8B). Interestingly, nuclear capsids were also labeled by this antibody, but lacked epitopes of the middle portion of pUL36. These results suggest that only a C-terminal fragment of pUL36 was associated with nuclear capsids. Similar to pUL36, surface exposure of pUL37GFP was highest on 0.1 and 0.5 M, reduced to 25% on 1 M KCl, and absent on nuclear capsids (Fig. 8B).

For comparison, we also determined the labeling efficiency for the outer tegument proteins VP16, VP13/14 and VP22 that were detected best on the surface of 0.1 M KCl capsids, and reduced on 0.5, 1 M and nuclear capsids (Fig. 8C). Of those, the VP22 epitopes showed the strongest difference between 0.5 and 1 M KCl capsids. Together with the immunoblot and mass spectrometry analysis, these immunoelectron microscopy-labeling patterns were most consistent with a gradual removal of the major tegument proteins from viral capsids (c.f. Fig. 3). In contrast, the minor capsid protein pUL25 was at the vertices of all viral capsids and C capsids in similar amounts, but reduced on B capsids (Fig. 8D). These results suggest that the asymmetric tegument cap [26] did not tightly cover the entire capsid surface, since it did not prevent access of antibodies, and presumably also not of host proteins to major capsid proteins.

In summary, increasing the salt concentration resulted in an increased removal of outer tegument proteins and destroyed epitopes of the inner tegument proteins pUL36 and pUL37GFP, although these proteins were still detected on 1 M KCl capsids by immunoblot and mass spectrometry analysis (Fig. 3). C-terminal epitopes or fragments of pUL36 that were detected on nuclear capsids were also denatured or even dissociated from 1 M KCl capsids. Thus, the surface analysis by immunoelectron microscopy revealed clear differences between 0.5 and 1 M KCl capsids that had remained elusive by immunoblot or mass spectrometry analysis suggesting a salt-induced conformational change of the inner tegument layer that abrogated kinesin-1, but not dynein or dynactin recruitment.

## Discussion

**Figure 9:**
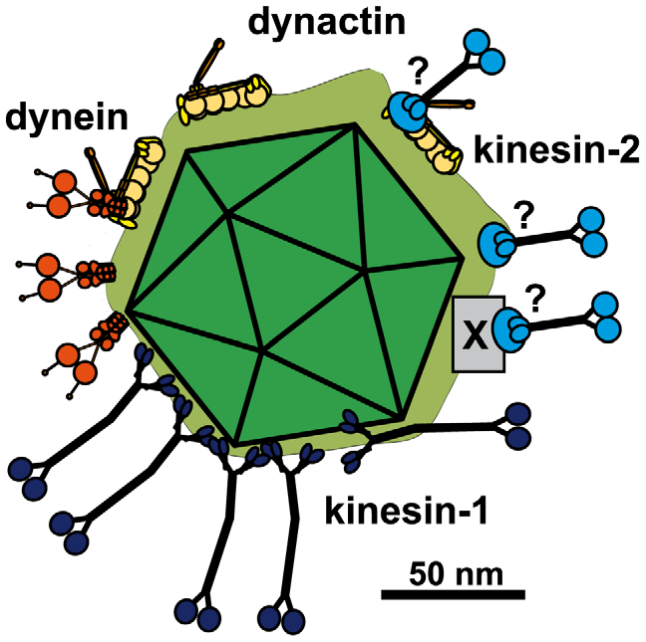
Scale drawing of a HSV1 viral capsids exposing inner tegument proteins and interacting with MAPs. Tegumented (light green) HSV1 capsid (dark green) with three bound dynein (orange), three dynactin (yellow), five kinesin-1 (dark blue) and 3 kinesin-2 (light blue). The scheme has been drawn to scale and assuming that MT binding domains of the MAPs point away from the capsid to enable microtubule binding. Dynein, dynactin and kinesin-1 can interact directly and independently of each other with the capsids. Individual capsids could recruit several copies of a MAP, and different MAPs at once. Dynein and Kinesin-2 may either bind directly to tegument proteins or indirectly via dynactin. Furthermore, kinesin-2 may also utilize another, unknown host factor (X). Scale bar: 50 nm.

We used five different capsid types and mammalian brain cytosol to analyze the interaction of HSV1 capsids with host MT motors. Starting with nuclei of infected cells or extracellular virions, and varying the salt concentration during virion lysis, we generated capsids with different protein composition and different surface features without impairing their overall architecture. Using immunoblot and quantitative immunoelectron microscopy, we show that dynein, dynactin, kinesin-1 and kinesin-2 bound specifically to viral capsids, but not to nuclear capsids (summarized in Fig. 3 & 9). Presumably, entire MAP complexes, and not just individual subunits, associated with the viral capsids, since we could detect several subunits of each MAP. In contrast, GST or other cytosolic host proteins such as tau, amphiphysin or LC3 were not recruited.

**Dynein** colocalizes with inbound cytosolic HSV1 capsids [33], [34]. Furthermore, outbound capsids might use dynein for transport to the trans-Golgi network, where HSV1 capsids acquire their final envelope [4], [30], [37], [38], [39; Döhner, Sodeik & Bauerfeind, unpublished observations]. Our experiments with purified motors showed that dynein can bind directly - independent of other host factors - to tegumented capsids. Similarly **dynactin** also bound directly and independently of other host factors to viral capsids. This is different from many host cargos that utilize dynactin as an adaptor to recruit dynein [16], [18], [58]. However, destroying the dynactin complex by an artificial excess of its subunit dynamitin reduces nuclear targeting of HSV1 capsids *in vivo* as well as the number of motile capsids and the transport velocity *in vitro* [23], [34]. Thus, dynactin may rather enhance dynein processivity than function as an adaptor to recruit dynein onto capsids, as has also been described for some host cargos [16], [18], [58].

Dynactin also enhances the processivity of **kinesin-2**, it is a cofactor for both, dynein and kinesin-2 mediated transport of *Xenopus* melanosomes, and it interacts directly with kinesin-2 [59], [60]. KIF3A, one of the heavy chains of kinesin-2, interacts with pORF45, a tegument protein of the gammaherpesvirus KSHV that has no homolog in alphaherpesviruses. Inhibition of kinesin-2 activity by siRNA gene silencing reduces viral yields of KSHV in lymphoma cells, but not of HSV1 in epithelial cells [61]. Thus, so far, there is no evidence of a role for kinesin-2 in the life cycle of alphaherpesviruses. Nevertheless, tegumented, but not nuclear HSV1 capsids specifically recruited kinesin-2, either directly via a tegument protein or via its interaction with dynactin or another host factor (Fig. 9). Kinesin-2 may either facilitate intracellular transport in epithelial cells in a non-essential manner, or catalyze axonal transport of outbound particles in neurons.

**Kinesin-1** also bound directly in the absence of other host factors to 0.5 M KCl capsids. This differs from many membranous host cargos that recruit kinesin-1 via adaptors such as huntingtin-associated protein 1, sunday driver or amyloid precursor protein [9], [62], [63]. However, in contrast to tubulin and several proteins of the actin cytoskeleton, such adaptors or other MAP subunits have not been detected in purified herpesvirus particles [20], [64]. Kinesin-1 may catalyze HSV1 capsid transport from the MTOC to the nucleus, it colocalizes with outbound HSV1 particles in axons, and it may mediate axonal transport of progeny cytosolic capsids [4], [40], [49]. Inhibiting kinesin-1 but not kinesin-2 by siRNA gene silencing or overexpression of dominant-negative protein domains reduced nuclear targeting of inbound HSV1 capsids in epithelial cells (Janus & Sodeik, in preparation).

The **number of MT motors** is unknown for viral cargos, but on host cargos ranges from 2 to 10 or even more [reviewed in 7], [65]. Just one dynein or kinesin-1 motor is sufficient to transport synthetic beads over long distances *in vitro*, whereas at high dynein-dynactin concentrations such beads stall at MT intersections [66]. Cargo driven by too many motors seems more likely to get trapped in a fruitless tug-of-war between motors interacting with different MTs. Each reaction contained viral capsids derived from 5×10^8^ PFU/60 µL with about 10 to 60 DNAse protected genomes/PFU [42]. Taking this number of genomes as the lowest estimate, the viral capsids were within a concentration of 0.1 to 0.8 nM. Based on immunoblots and comparing with protein concentrations of the MAP preparations, we estimate that our cytosolic extracts contain about 30 nM dynein, 40 nM dynactin, and 190 nM kinesin-1. Thus, there were about 35 to 300 molecules dynein, 50 to 400 molecules dynactin, and 240 to 1900 molecules of kinesin-1 present per capsid. Compared to the cytosol loading control, about 1 to 20% of the total MAPs were co-sedimented with viral capsids treated with 0.5 M KCl. These rough estimations suggest that in our assays, a capsid recruited 0.3 to 60 copies of dynein, 0.5 to 80 copies of dynactin, and 2.5 to 380 copies of kinesin-1.

On thawed cryosections of cells, protein-A gold labels antibodies directed against dynein on about 13% of inbound cytosolic HSV1 capsids [33]. The labeling efficiency for this technique is estimated to detect 5 to 10% of the antigens present [47], [67]. Thus, it seems reasonable to assume that, in cells, all incoming capsids are associated with dynein. Here we labeled for host factors bound to capsids *in vitro* which should be even better accessible to antibodies, although the antibodies and protein-A gold may still preferentially label the apical capsid surfaces over those facing the substrate. Our immunoelectron microscopy demonstrated that at least 20% of the capsids accommodated more than one copy of dynein, and about 10% more than one dynactin or kinesin-1 complex.

Since dynein and dynactin, but not kinesin-1, bound to 1 M KCl capsids (Fig. 3, Supplementary Table S1), and given that purified dynein and dynactin bound independently of each other, these 3 MAPs seem to associate with different surface features or domains on 0.5 M KCl capsids. As nuclear capsids did not bind MAPs, the tegument and not the capsid proteins must contain the **viral receptors for microtubule motors**. Capsids derived from HSV1 knock-out strains showed that VP26, pUS11 or VP11/12 do not contribute to the generation of the viral motor receptors that we analyzed here. Furthermore, PrV does not encode a homolog of US11, and HSV1 mutants lacking VP26 or pUS11, as well as PrV lacking VP26, pUL14, VP11/12 or VP22 are not impaired in nuclear targeting of capsids [42], [68], [69; Janus & Sodeik, in preparation]. Thus, these viral proteins are less likely to contribute to the generation of viral motor binding domains.

Interestingly, capsids derived from a HSV1 mutant lacking VP11/12 bound even more MAPs than wild-type. As dynein, dynactin, kinesin-1 or kinesin-2 are not present in complete virions [64], the acquisition of outer tegument proteins during assembly may gradually compete for MAP binding. Since outer tegument proteins associate with viral envelope proteins [30], [37], MAPs may even need to dissociate prior to secondary envelopment. A reverse reaction seems to occur during cell entry. Upon viral fusion, the bulk of tegument proteins detaches from incoming HSV1 and PrV capsids and remains bound to the viral envelope, thereby exposing the underlying capsid-associated inner tegument proteins [26], [33], [70], [71], [72]. We suppose that treating virions with TX-100 and 0.5 M KCl results in a similar separation of the outer from the inner tegument layer, and thus enables access of cytosolic host factors to capsid-associated viral motor receptors. This notion is supported by the low binding of MAPs to capsids treated with 0.1 M KCl that still contain high amounts of the tegument proteins vhs, pUL11, ICP4, ICP34.5, VP11/12, VP13/14, VP16, and VP22. We therefore classified proteins that were abundant on 0.1 M capsids, but reduced by treatment with 0.5 or 1.0 M KCl as outer tegument proteins (Fig. 3; Supplementary Table S1). This outer tegument rather prevented than mediated a recruitment of MT motors to herpesvirus capsids.

The 0.1 and 0.5 M KCl treated capsids contained other proteins in similar amounts that we consider inner tegument components: pUS3, pUL36, pUL37, ICP0, pUL14, pUL16, and pUL21 (Fig. 3, Supplementary Table S1). While pUL21 is not required for retrograde spread of PrV, the tegument is not properly assembled in a PrV mutant lacking UL21 [69], [73], [74]. Thus, binding assays using capsids derived from such mutants alone would not suffice to demonstrate that pUL21 acts as a viral receptor for motors. pUL36, pUL37, or pUL21 may interact with kinesin-1 or at least contribute to the formation of a viral kinesin-1 receptor, since these proteins were reduced or at least some of their epitopes were denatured by treating viral capsids with 1 M KCl (Fig. 3, Supplementary Table S1).

Considering all HSV1 structural proteins, **pUL36 and pUL37** are likely candidates to either directly enlist motors to capsids, or to recruit other tegument proteins that function as viral motor receptors. pUL37 interacts directly with the capsid protein VP26, and with pUL36 that is linked via pUL25 to capsids [75], [76], [77]. pUS3, pUL36, and pUL37GFP remain associated with incoming capsids during nuclear targeting; and HSV1 and PrV mutants lacking pUL36 or pUL37 are impaired in intracellular transport [43], [69], [71], [78], [79], [80], [81], [82], [83; Schipke & Sodeik, in preparation]. We show here that pUL36 and pUL37GFP were exposed on the surface of 0.5 M KCl treated capsids, and that epitopes of pUL36 and pUL37 were altered by increasing KCl concentration from 0.5 to 1 M which also inactivated the receptor binding site for kinesin-1 (Fig. 3, Supplementary Table S1).

Unfortunately, capsids of HSV1 or PrV mutants lacking pUL36 or pUL37 cannot be isolated from extracellular virions, since cytosolic capsids are not enveloped, and no virions are assembled [43], [78], [79]. Therefore, binding assays with capsids derived from such mutants require new purification protocols to isolate sufficient amounts of cytosolic progeny capsids not contaminated by the more abundant nuclear capsids [43]. Considering the multivalent interactions among several tegument proteins [30], [37], [77], the lack of pUL36 or pUL37 may result in an altered tegument organization, as has already been shown for mutants lacking pUL21 [69], [73], [74]. Future binding studies with such mutant capsids will therefore require a thorough investigation of their molecular tegument architecture.

The capsid **transport direction** to the cell center or periphery during cell entry and egress must be regulated. Our data suggest that dynein and kinesin-1 bind to distinct surface features on 0.5 M KCl capsids, since treatment with 1 M KCl specifically inactivated kinesin-1 but not dynein binding. A similar modification of the tegument surface may also occur *in vivo* and change the affinity of capsids for motors, e.g. during cell entry when pUL36 is cleaved, or tegument proteins are phosphorylated [70], [84]. Thus, an “exclusive” presence of either minus- or plus-end directed motors on viral capsids of different protein composition may regulate their transport direction [8], while such a mechanism seems to be less used for host cargos [6], [7], [9], [85]. Time-lapse microscopy shows that HSV1 and PrV particles quickly change transport directions *in vivo* suggesting that motors of opposing directionality are present simultaneously on viral particles. Nuclear targeting of inbound and egress of outbound PrV particles can occur simultaneously within the same cell, suggesting that there is no overall inhibition or stimulation of host factors controlling transport direction during herpesvirus infection [32]. However, in many live cell microscopy experiments, transport of cytosolic capsids cannot be distinguished from transport of virions within host membranes [31], [32], [86; Janus & Sodeik, in preparation].

Our biochemical single-particle analysis shows that at least 10% of all capsids had bound the both motors dynein and kinesin-1 simultaneously. Thus, it seems likely that also in cells individual cytosolic capsids can recruit the entire transport machinery of dynein, dynactin, and kinesin-1 (Fig. 9). Therefore, the transport direction of HSV1 capsids does not seem to be regulated by an exclusive presence of either plus- or minus-end directed motors. The bound motors rather either engage in a tug-of-war to determine the direction of net transport, or the activities of the bound motors are coordinately regulated. For example, an alternating activation of either plus- or minus-end directed motors e.g. by host or viral kinases or binding of cellular inhibitors could mediate fast changes in transport direction.

**Future studies** with such biochemical assays will provide further insights into the regulation of essential virus-host interactions, for example by adding, depleting, or inhibiting specific host factors such as kinases. Furthermore, the functions of structural viral proteins can be elucidated even if their mutation prevents virus formation or cell culture models are lacking. Recombinant proteins, protein fragments, or peptides derived from viral genes can be tested for their ability to compete with particle-associated viral proteins for the recruitment of host factors. Such assays will identify viral structures that are required for motor binding and capsid nuclear targeting, and that therefore should be maintained in viral gene therapy vectors. These structural features or domains may even be added to artificial nano carriers or other vectors to be exploited for human therapeutic gene and cell therapy.

## Materials and Methods

### Viruses, cells and antibodies

HSV1 strains F (ATCC VR-733), 17^+^ [87], HSV1(KOS)-ΔVP26 (HSV1-Kdelta26Z; from Prashant Desai, Johns Hopkins University, Baltimore, USA; [68]), HSV1(F)-ΔUS11 (HSV1-G6/166; from Richard Roller, University of Iowa, Iowa, USA; Janus & Sodeik, in preparation), HSV1(F)-ΔVP11/12 (R[F] UL46Δ3.1; from Jennifer L.C. Mc Knight & Tom Kristie, NIH, Bethesda, USA; [88], [89]), and HSV1(17^+^)-pUL37GFP (from Frazer Rixon, MRC Virology Unit, Glasgow, UK; [90]) were propagated in BHK-21 and titrated on Vero cells [42]. Antibodies directed against viral or host proteins are listed in the supplement (Tables S2, S3).

### Viral capsids

Particles sedimented from the medium of infected cells, corresponding to approximately 5×10^8^ PFU of HSV1, were left untreated, or incubated with 0.5 mg/ml trypsin (Sigma-Aldrich, Germany) at 37°C for 1 h and with 5 mg/ml trypsin inhibitor from soybean (Fluka, Switzerland) for 5 min on ice. Virions were lysed for 30 min on ice by adding an equal volume of twofold lysis buffer (2% TX-100, 20 mM MES, 30 mM Tris, pH 7.4, 20 mM DTT, 20 µg/ml antipain, 4 µg/ml bestatin, 4 µg/ml pepstatin, 4 µg/ml aprotinin, 20 µg/ml E-64, 4 µg/ml leupeptin, 320 µg/ml phenylmethylsulfonylfluoride) containing KCl to achieve final concentrations of 0.1 M, 0.5 M or 1 M.

Capsids were separated from solubilized membrane and tegument proteins by layering the extract on top of a 20% (w/v) sucrose cushion in 30 mM MES and 20 mM Tris, pH 7.4 with 10 mM DTT, protease inhibitors as above and the respective KCl concentrations and sedimentation at 50 krpm for 15 min at 4°C in a TLA100.2 Beckman rotor. Supernatants and cushions were carefully removed, and the pellets were resuspended in BRB80 (80 mM PIPES, pH 6.8, 12 mM MgCl_2_, 1 mM EGTA) with 10 mM DTT, protease inhibitors including 1 mg/ml soybean trypsin inhibitor and 0.1 U/µl protease-free DNase I (Promega, USA) and 100 µg/ml protease-free RNase (Roth GmbH, Germany). After incubation for 30 min at 37°C and over night at 4°C, capsids were repelleted for 8 min at 50 krpm in a TLA100.2 rotor at 4°C, and resuspended in cytosol or buffer by tip sonification on ice 3 times for about 10 seconds at 40 W.

### Nuclear capsids

BHK-21 cells were infected with 0.01 to 0.02 PFU/cell for 2 to 3 days until the cells detached from culture flasks. Infected cells were collected by sedimentation, washed once with MNT buffer (30 mM MES, 20 mM Tris, pH 7.4, 100 mM NaCl), snap frozen and stored at −80°C. Nuclear capsids were prepared as described previously [23], [91], diluted in three volumes TNE (20 mM Tris, pH 7.5, 500 mM NaCl, 1 mM EDTA) with 10 mM DTT and protease inhibitors, and sedimented in BSA-coated centrifuge tubes at 50 krpm in a Beckman TLA100.2 rotor at 4°C for 15 min. The pellets were resuspended in BRB80 buffer with 10 mM DTT, 1 mg/ml soybean trypsin inhibitor, protease inhibitors, 100 µg/ml RNase (Roth, Germany) and 0.1 U/µl DNase I (M6101, Promega, USA) and treated as described for viral capsids.

### Cytosol and microtubule motors

Pig brain cytosol was prepared according to [92]. Brains of Pieton pig were homogenized in one volume extraction buffer (50 mM PIPES, 50 mM HEPES, pH 7.0, 2 mM MgCl_2_, 1 mM EDTA) with 1 mM DTT and protease inhibitors with a Potter-Elvejehm homogenizer. Cellular debris was sedimented at 24,000 g for 30 min at 4°C, and the supernatant was snap-frozen in liquid nitrogen and stored at −80°C. Directly prior to binding experiments, 1/20 volume of an ATP-regenerating system (150 mM creatine phosphate, 20 mM ATP, 20 mM MgCl_2_, 2 mM EGTA, pH 7.7) was added to the extracts to induce release of membrane-bound motors [93]. After sedimentation of membranes at 55 krpm and 4°C for 30 min in a TLA100 rotor, the membrane-free supernatant containing soluble cytosolic factors was collected. After addition of 10 U/ml apyrase (Sigma-Aldrich, Germany), and incubation at room temperature for 10 min to deplete endogenous ATP, the cytosol was diluted in acetate buffer (10 mM PIPES, pH 7.4, 100 mM potassium acetate, 3 mM magnesium acetate, 5 mM EGTA) with 5% (w/w) sucrose, 1 mg/ml BSA, 10 mM DTT, 50 µM nocodazole and protease inhibitors. Cytosolic extracts prepared by this protocol were devoid of membranes, and thus of cellular transport vesicles (Fig. S2). Dynein and dynactin were purified from bovine brain [94] and kinesin-1 from pig brain [95], and the purity of the these preparations were confirmed by SDS-PAGE (not shown).

### Co-sedimentation of host factors with HSV1 capsids

Viral capsids or nuclear capsids were resuspended in 60 µl cytosol (0.25 mg/ml protein for dynein and dynactin, 0.75 mg/ml for kinesin-1 and kinesin-2) or buffer. After 1 h incubation at 0°C (Fig. 2 & 4A) or 37°C (Fig. S1, 4B, 5), the capsids were either sedimented through a 30% (w/v) sucrose cushion in acetate buffer by centrifugation at 50 krpm for 15 min in a Beckman TLA100 rotor, or directly subjected to immunogold-labeling and electron microscopy. Pellets were washed once in acetate buffer, spun for 8 min at 50 krpm in a TLA100 rotor and analyzed by SDS-PAGE and immunoblotting. Controls lacking cytosol revealed some but not all preparations of viral capsids already contained dynein and dynactin in varying amounts (Fig. S1B, compare lane 1 with lane 3). To remove such host factors, we first incubated the extracellular particles with trypsin (Fig. S1A). After adding trypsin inhibitor and lysis buffer, we then isolated viral capsids. A large fraction of the major capsid proteins VP5, VP19c and the tegument protein VP13/14 was protected by the viral membrane of intact virions, and protected from tryptic cleavage (Fig. S1B, compare lane 2 and 4). Such capsids contained little dynein and dynactin (Fig. S1B, lane 8), but were still able to recruit new MAPs from the cytosol (Fig. S1B, lane 10). Without viral capsids, MAPs by themselves did not sediment (Fig. S1B, lanes 5 and 6).

### SDS-PAGE and immunoblot

Protein samples were separated on linear 7.5 to 18% or 5 to 15% SDS-PAGE, transferred onto nitrocellulose membrane (Pall Corporation, USA) and detected using specific primary antibodies (c.f. Tables S2,S3) and secondary antibodies coupled to horse-radish peroxidase (Dianova or Pierce-Perbio Science, Germany) for ECL detection (SuperSignal West Femto Maximum Sensitivity Substrate, Pierce, Germany).

### Negative staining and immunoelectron microscopy

After adsorption on 400 mesh formvar- and carbon-coated copper grids (Stork Veco, The Netherlands) and blocking unspecific protein binding sites with 10 mg/ml BSA in PBS, the samples were labeled with primary mouse antibodies and rabbit anti-mouse IgG (Cappel™, MP Biomedicals, USA) or just with primary rabbit antibodies followed by protein-A gold (10 nm; Cell Microscopy Center, Utrecht School of Medicine, The Netherlands). After washing with PBS and distilled water, preparations were negatively contrasted using 2% (w/v) uranyl acetate (Merck, Germany), and analyzed with an EM10CR transmission electron microscope (Carl Zeiss AG, Germany) at 80 kV. For quantification of MAPs bound to capsids, samples were prepared from capsids incubated with cytosol or with buffer as a negative control. The number of gold particles per capsid incubated with buffer was considered background labeling and subtracted from the labeling on capsids that had been incubated with cytosol. For analysis of viral proteins, a similar background subtraction was performed with capsids labeled in the absence of a primary antibody. Background labeling was similar in both approaches and ranged from 0.3–0.5 gold/capsid without cytosol and 0.3–0.7 gold/capsid without a primary antibody. For negative staining without labeling, grids with adsorbed virus particles were washed three times with PBS, incubated in distilled water for 10 min, washed in distilled water and negatively contrasted by incubation with 10 µl 2% (w/v) uranyl acetate (Merck, Germany).

### Mass spectrometry

SILAC was performed with slight modifications as described previously [51]. BHK-21 cells were cultured for three passages in DME-nutrient mixture F12 Ham medium (Sigma-Aldrich D-9785) supplemented with 5% (v/v) dialyzed FCS and either deuterated (L-leucine-5,5,5 d_3_; Sigma-Aldrich; 99 atom% D) or conventional protonated L-leucine. The isotope exchange in the deuterated cells was confirmed by mass spectrometry. Isotope labeled cells and media were then used to prepare deuterated and protonated virus stocks of HSV1 strain F. Deuterated virions were further purified by sedimentation at 50,000 g for 2 h at 4°C through a linear 10% to 40% (w/v) nycodenz gradient in MKT buffer, and collection of the light scattering band. Protonated virions were sedimented and protonated capsids were isolated from them as described above. 5 µg protonated virions or capsids and 5 µg deuterated reference virions were mixed and separated by SDS-PAGE. After staining with Coomassie Blue, all visible bands were processed and mass spectra were registered with an Ultraflex MALDI-TOF (matrix-assisted laser desorption/ionization - Time-Of-Flight) mass spectrometer with TOF/TOF option (Bruker Daltonics, Bremen, Germany) as described in [55]. Proteins were identified by query of an in-house database representing the proteome of HSV1 strain 17^+^ as compiled from the Uniprot database in October 2009 [96]. Proteins identified with a significant score (95% significance level, corresponding to a MOWSE score of 33 in the configuration used) were quantified using the in-house software AmaDeuS [97]. A minimum of three different peptide pairs were used for the relative quantification, with the exception of VP26 that yielded only one leucine-containing tryptic peptide (Table S4). Mean values of intensity ratios of all proteins were normalized to the relative abundance of the VP5 protein in the same sample to account for varying amounts of capsids in each capsid preparation.

## Supporting Information

Table S1 Characterization of tegumented, viral HSV1 capsids. The MAP binding (Fig. 2) to the three viral capsid types treated with 0.1, 0.5 or 1 M KCl and their inner and outer tegument organization (Figs. 6,7,8) have been analyzed by immunoblot (IB), quantitative mass spectrometry (MS), and quantitative immunoelectron microscopy (IEM). While IB and MS indicate the amount of different tegument proteins on the capsids, the IEM determines to what extend such tegument proteins were accessible on the capsid surfaces to antibodies or host factors. Please note that nuclear capsids did not bind to MAPs and contained very little tegument (c.f. Figs. 2, 6, 7, 8; C capsids not shown in this table). For further comparative analysis, we normalized the results of the IB, MS and IEM for the viral capsids such that the amount of a given MAP or a tegument protein on the capsids with the highest amount was set to 100%, and recalculated accordingly for the other capsid types (% of highest). These results are also graphically displayed in Fig. 3. Please note that in contrast to MS and IEM, the IB data were not quantitative. Instead, we estimated the amount of the respective proteins based on the band intensities into 4 classes: absent (0), minor (+), major (++) or highest (+++) amounts.
(0.11 MB DOC)

Table S2 List of primary antibodies directed against HSV1 proteins.
(0.06 MB DOC)

Table S3 List of primary antibodies directed against host proteins or tags and secondary antibodies.
(0.05 MB DOC)

Table S4 List of peptides identified by mass spectrometry. The protein composition of HSV1 capsids, virions sedimented from cell culture supernatants and gradient purified virions were analyzed by quantitative mass spectrometry. Under the experimental conditions, Mowse scores exceeding 33 are significant at the 95% level. Scores and sequence coverages of the experiment with the highest score are given. *Calculated on basis of the full length pUL36.
(0.53 MB DOC)

Figure S1 Preparation of HSV1 capsids lacking host proteins. **A.** Extracellular particle preparations contain virions (dark+light green), light particles consisting of a membrane and tegument but no capsid (light green), unenveloped and/or broken capsids (dark green) with associated motors and other host cell proteins (small, colored circles). The extracellular particles sedimented from the medium of infected cells (MP, medium pellet) were therefore incubated with trypsin for 1 h, and subsequently with an excess of trypsin inhibitor to remove pre-bound host factors while protecting viral proteins by the viral envelope. Such pretreated particles were then lysed with a buffer containing TX-100 and different amounts of KCl to isolate viral capsids by sedimentation through sucrose cushions (c. f. Fig. 1). **B**. Immunoblot analysis of viral particles untreated or incubated with trypsin and of their ability to recruit MAPs. Extracellular particles of HSV1(F) corresponding to 5×10^8^ pfu were either left untreated in buffer (lanes 1, 3, 7, 9) or incubated with trypsin (lanes 2, 4, 8, 10) prior to addition of trypsin inhibitor and lysis with TX-100 in the presence of 0.5 M KCl. Viral capsids were sedimented, incubated in buffer (lanes 1 to 4, 7, 8) or 0.75 mg/ml pig brain cytosol (lanes 9, 10), and sedimented through sucrose cushions. The resulting pellets were analyzed by immunoblot using antibodies against against dynein (lanes 1 to 4, MAB1618 against intermediate chain; lanes 5 to 10, anti-LIC2 against light intermediate chain), and dynactin (lanes 1 to 4, mAb anti-p50; lanes 5 to 10, mAb3F2.3 against CapZβ). The amount of capsids in each sample was estimated by labeling the major capsid proteins VP5 (pAb NC-1) or VP19c (pAb NC-2) or the outer tegument protein VP13/14 P26 (pAb R220). As controls, the cytosol was also directly analyzed (lane 5), or sedimented in the absence of capsids (lane 6). Please note that the two different HSV1(F) preparations (lanes 1 and 2 versus lanes 3 and 4) contained different amounts of pre-bound host factors (lanes 1 and 3) that could be reduced in both cases by trypsin treatment (lanes 2 and 4). A third HSV1(F) preparation (lanes 7 to 10) contained minor amounts of pre-bound host factors that was reduced by the trypsin treatment (compare lanes 7 with 8). The viral capsids isolated from this trypsin-treated preparation of extracellular particles recruited additional host factors after the incubation with cytosol in both cases, without prior trypsin treatment (compare lanes 7 with 9) after trypsin treatment (compare lanes 8 with 10). These blots show one of three independent experiments yielding similar results.
(1.12 MB TIF)

Figure S2 The mammalian cytosol fraction does not contain membranes. Pig brain extract or buffer were incubated with 1 µM DiIC_18_ (Molecular Probes, Eugene, USA) for 30 min at room temperature and subsequently centrifuged as described before (Wolfstein et al. 2006). Labeled membranes in the brain extract, buffer as a control, the pellet and the supernatant of the centrifugation were analyzed by fluorescence microscopy. Only traces of membranes were detected in the cytosolic supernatant.
(0.18 MB TIF)
