# Supplementary material for: Plus- and Minus-End Directed Microtubule Motors Bind Simultaneously to Herpes Simplex Virus Capsids Using Different Inner Tegument Structures
Source: PLoS Pathog. 2010 Jul 8;6(7):e1000991. doi: 10.1371/journal.ppat.1000991 (PMC2900298; doi:10.1371/journal.ppat.1000991)
Supplement: Table S1 — Characterization of tegumented, viral HSV1 capsids. The MAP binding (Fig. 2) to the three viral capsid types treated with 0.1, 0.5 or 1 M KCl and their inner and outer tegument organization (Figs. 6,7,8) have been analyzed by immunoblot (IB), quantitative mass spectrometry (MS), and quantitative immunoelectron microscopy (IEM). While IB and MS indicate the amount of different tegument proteins on the capsids, the IEM determines to what extend such tegument proteins were accessible on the capsid surfaces to antibodies or host factors. Please note that nuclear capsids did not bind to MAPs and contained very little tegument (c.f. Figs. 2, 6, 7, 8; C capsids not shown in this table). For further comparative analysis, we normalized the results of the IB, MS and IEM for the viral capsids such that the amount of a given MAP or a tegument protein on the capsids with the highest amount was set to 100%, and recalculated accordingly for the other capsid types (% of highest). These results are also graphically displayed in Fig. 3. Please note that in contrast to MS and IEM, the IB data were not quantitative. Instead, we estimated the amount of the respective proteins based on the band intensities into 4 classes: absent (0), minor (+), major (++) or highest (+++) amounts. (0.11 MB DOC) [file ppat.1000991.s001.doc]

**Table S1: Characterization of HSV1 capsids interacting with MAPs.**

| **Protein** | **Method** | **0.1 M** | **0.5 M** | **1 M** | **nuclear B** |
| --- | --- | --- | --- | --- | --- |
| **Microtubule associated protein** | | **-** | **+++** | **++** | **-** |
| dynein | IB | - | +++ | +++ | - |
|  | IEM [%] | 33 | 100 | 98 | 0 |
| dynactin | IB | - | +++ | ++ | - |
|  | IEM [%] | 3 | 100 | 66 | 0 |
| kinesin-1 | IB | - | +++ | - | - |
|  | IEM [%] | 4 | 100 | 19 | 0 |
| kinesin-2 | IB | + | +++ | ++ | - |
|  | IEM [%] | 0 | 100 | 43 | 35 |
| **Ruled out by HSV1 deletion mutants** | | | | | |
| VP26 | IB | +++ | +++ | +++ | ++ |
|  | MS [%] | 100 | 101 | 102 | 125 |
| pUS11 | IB | + | - | - | - |
| VP11/12 | MS [%] | 374 | 383 | 281 | - |
| **Outer tegument protein** | | **+++** | **++** | **+** | **-** |
| pUL41 (vhs) | IB | +++ | ++ | + | - |
| pUL11 | IB | +++ | + | + | - |
| ICP4 | IB | +++ | ++ | + | - |
| ICP34.5 | IB | +++ | ++ | ++ | - |
| VP13/14 | IB | +++ | ++ | + | - |
|  | MS [%] | 124 | 65 | 12 | - |
|  | IEM [%] | 100 | 71 | 56 | 32 |
| VP16 | IB | +++ | ++ | ++ | - |
|  | MS [%] | 72 | 41 | 19 | - |
|  | IEM [%] | 100 | 89 | 65 | 15 |
| VP22 | IB | +++ | ++ | + | - |
|  | MS [%] | 143 | 55 | 23 | - |
|  | IEM [%] | 100 | 72 | 24 | 6 |
| **Inner tegument protein** | | **+++** | **+++** | **+++/-** | **-/+** |
| pUS3 | IB | +++ | +++ | +++ | + |
|  | IEM [%] | 100 | 90 | 75 | 19 |
| pUL36 | IB | +++ | ++ | ++ | -/+ |
|  | MS [%] | 20 | 28 | 18 | - |
|  | MS - Nterm [%] | 161 | 189 | 183 | - |
|  | IEM - middle Ab [%] | 76 | 100 | 81 | 1 |
|  | IEM - Cterm Ab [%] | 58 | 100 | 2 | 36 |
| pUL37 | IB (GFP) | +++ | +++ | ++ | - |
|  | MS [%] | 48 | 49 | 21 | - |
|  | IEM [%] (GFP) | 100 | 94 | 32 | 1 |
| ICP0 | IB | +++ | +++ | ++ | - |
| pUL14 | IB | +++ | +++ | +++ | + |
| pUL16 | IB | +++ | ++ | ++ | - |
|  | MS [%] | 42 | 45 | 21 | - |
| pUL21 | MS [%] | 50 | 55 | 19 | - |

The MAP binding (Fig. 2) to the three viral capsid types treated with 0.1, 0.5 or 1 M KCl and their inner and outer tegument organization (Figs. 6,7,8) have been analyzed by immunoblot (IB), quantitative mass spectrometry (MS), and quantitative immunoelectron microscopy (IEM). While IB and MS indicate the amount of different tegument proteins on the capsids, the IEM determines to what extend such tegument proteins were accessible on the capsid surfaces to antibodies or host factors. Please note that nuclear capsids did not bind to MAPs and contained very little tegument (c.f. Figs. 2, 6, 7, 8; C capsids not shown in this tabl*e*).

For further comparative analysis, we normalized the results of the IB, MS and IEM for the viral capsids such that the amount of a given MAP or a tegument protein on the capsids with the highest amount was set to 100%, and recalculated accordingly for the other capsid types (% of highest). These results are also graphically displayed in Fig. 3. Please note that in contrast to MS and IEM, the IB data were not quantitative. Instead, we estimated the amount of the respective proteins based on the band intensities into 4 classes: absent (0), minor (+), major (++) or highest (+++) amounts.
