# Supplementary material for: Plus- and Minus-End Directed Microtubule Motors Bind Simultaneously to Herpes Simplex Virus Capsids Using Different Inner Tegument Structures
Source: PLoS Pathog. 2010 Jul 8;6(7):e1000991. doi: 10.1371/journal.ppat.1000991 (PMC2900298; doi:10.1371/journal.ppat.1000991)
Supplement: Table S2 — List of primary antibodies directed against HSV1 proteins. (0.06 MB DOC) [file ppat.1000991.s002.doc]

**Table S2 : List of antibodies directed against HSV1 proteins.**

| **Antigen** | **Antibody name** | **Type**  **Species** | **Reference and Source** |
| --- | --- | --- | --- |
| **HSV1 capsid protein** | | | |
| VP5 (band from gel) | NC-1 | pAb rabbit | [100], G. Cohen & R. Eisenberg (U of Pennsylvania, Philadelphia, USA) |
| VP19c (band from gel) | NC-2 | pAb rabbit |
| VP26 (aa 95-112) | α-VP26 | pAb rabbit | [101], P. Desai (Johns Hopkins U, Philadelphia, USA) |
| pUL6 (full length, MBP-tag) | 1C9 | mAb mouse | [102,103], J. Brown (U of Viriginia, Charlottesville, USA) |
| pUL16 (C-term. 165 aa, GST-tag) | α-UL16 | pAb rabbit | [104], J. Baines (Cornell U, Ithaca) |
| pUL17 (aa 154-703, MBP-tag) | #203 | mAb mouse | [105], V. Preston (MRC Virology Unit, Glasgow, UK) |
| pUL25 (full length, GST-tag) | ID1 | pAb rabbit | [106], D. Tenney (Bristol-Myers-Sqibb, Wallingford) |
| **HSV1 tegument protein** | | | |
| pUS3 (aa 98-364, GST-tag) | α-pUS3 | pAb rabbit | [107], B. Roizman (U of Chicago) |
| M-pUL36 (aa 1408-2112) | #147 | pAb rabbit | [108], A. Helenius (ETH Zürich, Switzerland) |
| C-pUL36 (aa 3048-3057) | C-pUL36 | pAb rabbit | [109], R. Courtney (Pennsylvania State U, Hershey, USA) |
| pUL37 (C-term malE-tag) | 780 | pAb rabbit | [110], F. Jenkins (U of Pittsburgh, USA) |
| pUS11 (ß-gal-tag) | #28 | mAb rabbit | [111], R. Roller (U of Iowa) |
| pUL11 (aa 6-95, GST-tag) | α-pUL11 | pAb rabbit | [112], J. Baines (Cornell U, Ithaca, USA) |
| pUL14 (aa 58-219, GST-tag) | α-pUL14 | pAb rabbit | [113], J. Baines (Cornell U, Ithaca, USA) |
| pUL41 / vhs (full length, 6x His-tagged) | 11.388 | pAb rabbit | [114], R. Stewart (McArdle Lab. for Cancer Research, Madison, USA) |
| ICP0 (C-terminus) | 11060 | mAb mouse | [115,116], R. Everett (MRC Virology Unit, Glasgow, UK) |
| ICP4 | 58S | mAb mouse |
| ICP34.5 (aa146 – 263, GST-tag) | α-γ1-34.5 | pAb rabbit | [117], B. He (U of Illinois, Chicago, USA) |
| VP16 (aa 475-488) | SW7 | pAb rabbit | [118], D. Tenney (Bristol-Myers-Sqibb, Wallingford, USA) |
| VP13/14 (band from gel, full length) | R220 | pAb rabbit | [119], D. Meredith, U of York, UK) |
| VP22 (full length, GST-tag) | AGV30 | pAb rabbit | [120], G. Elliott (Imperial College, London, UK) |

mAb: monoclonal antibody. pAb: polyclonal antibody
