# Supplementary material for: Plus- and Minus-End Directed Microtubule Motors Bind Simultaneously to Herpes Simplex Virus Capsids Using Different Inner Tegument Structures
Source: PLoS Pathog. 2010 Jul 8;6(7):e1000991. doi: 10.1371/journal.ppat.1000991 (PMC2900298; doi:10.1371/journal.ppat.1000991)
Supplement: Table S3 — List of primary antibodies directed against host proteins or tags and secondary antibodies. (0.05 MB DOC) [file ppat.1000991.s003.doc]

**Table S3 : List of antibodies directed against host factors and tags.**

| **Antigen** | **Antibody name** | **Type species** | **Reference and Source** |
| --- | --- | --- | --- |
| **Host protein** | | | |
| dynein IC (aa 1-60 bovine brain) | MAB1618 | mAb mouse | Chemicon International Inc., USA |
| dynein LIC2 (rat brain, full length) | α-LIC2 | pAb rabbit | [121], R. Vallee (Columbia U, New York, USA) |
| dynactin p150^Glued^ (aa 3-202, human) | α-p150 | mAb mouse | BD Transduction Laboratories, Lexington, USA |
| dynactin p50 (aa 55-196, human) | α-p50 | mAb mouse | BD Transduction Laboratories, Lexington, USA |
| dynactin GST-CapZβ | mAb3F2.3 | mAb mouse | [122], developed by John Cooper and obtained from the Developmental Studies Hybridoma Bank under the auspices of the NICHD and maintained by the University of Iowa, Iowa City, USA. |
| kinesin-1 HC (N-term, bovine brain) | MAB1613 | mAb mouse | Chemicon International Inc., Temecula, USA |
| kinesin-1 LC (aa 1-50, bovine brain) | MAB1616 | mAb mouse | Chemicon International Inc., Temecula, USA |
| kinesin-2 KAP3A (aa 612-787, mouse) | α-KAP3A | mAb mouse | BD Transduction Laboratories, Lexington, USA |
| kinesin-2 KIF3A (sea urchin egg) | K2.4 | mAb mouse | Covance, Berkeley, USA |
| tau (aa 243-441, human) | α-tau | pAb rabbit | Dako Cytomation, Glostrup, Denmark |
| **Tag** | | | |
| GFP (full-length, *Aequorea victoria*) | JL-8 | mAb mouse | BD Living Colors, Franklin Lakes, USA |
| **Secondary antibodies** | | | |
| anti-rabbit, HRP-conjugated | #31460 | goat IgG | ThermoScientific, Rockford, USA |
| anti-mouse, HRP-conjugated | #1858413 | goat IgG | Pierce / ThermoScientific, Rockford, USA |
| anti-mouse for electron microscopy | α-mouse | rabbit IgG | Cappel, MP Biomedicals, Irvine, USA |

pAb: polyclonal antibody; mAb: monoclonal antibody
