# Supplementary material for: Plus- and Minus-End Directed Microtubule Motors Bind Simultaneously to Herpes Simplex Virus Capsids Using Different Inner Tegument Structures
Source: PLoS Pathog. 2010 Jul 8;6(7):e1000991. doi: 10.1371/journal.ppat.1000991 (PMC2900298; doi:10.1371/journal.ppat.1000991)
Supplement: Table S4 — List of peptides identified by mass spectrometry. The protein composition of HSV1 capsids, virions sedimented from cell culture supernatants and gradient purified virions were analyzed by quantitative mass spectrometry. Under the experimental conditions, Mowse scores exceeding 33 are significant at the 95% level. Scores and sequence coverages of the experiment with the highest score are given. *Calculated on basis of the full length pUL36. (0.53 MB DOC) [file ppat.1000991.s004.doc]

**Table S4: List of HSV1 peptides identified by quantitative mass spectrometry .**

| **protein** | **Uniprot ID** | **peptides identified** | **aa start** | **aa end** | **MOWSE score** | | **coverage** |
| --- | --- | --- | --- | --- | --- | --- | --- |
| **VP5** | P06491/  B9VQE6 | YAAAMVPTGSLLSTIEVASHR | 13 | 33 | | 296 | 52 |
|  |  | FPELAYMNEGR | 84 | 94 | |  |  |
|  |  | VQFEVHQPLIAR | 95 | 106 | |  |  |
|  |  | NVQAVLGAFER | 168 | 178 | |  |  |
|  |  | APPLALLLPMQR | 192 | 203 | |  |  |
|  |  | ATLVAELK | 217 | 224 | |  |  |
|  |  | EAVEAWLVDLTTATQPSVAVPR | 242 | 263 | |  |  |
|  |  | GRPVDGVLVTTAPIKQR | 271 | 287 | |  |  |
|  |  | VEDTEADVPVTYGEMVLNGANLVTALVMGK | 295 | 324 | |  |  |
|  |  | HLLEMQEEQLDLNR | 335 | 348 | |  |  |
|  |  | ADLVSIGEK | 365 | 373 | |  |  |
|  |  | IYAATNVPYPLVGAMDLTFVLPLGLFNPVMER | 384 | 415 | |  |  |
|  |  | FAAHAGDLVPAPGHPDPR | 416 | 433 | |  |  |
|  |  | DRQVLRLSLEHAIGTVCHPSLMNVDAAVGGLNR | 446 | 478 | |  |  |
|  |  | DPVEAANPYGAYVAAPAGPAADMQQLFLNAWGQR | 479 | 512 | |  |  |
|  |  | VVNGNLPLALCPAAFR | 577 | 592 | |  |  |
|  |  | VVNGNLPLALCPAAFRDARGLELGVGR | 577 | 603 | |  |  |
|  |  | NYPAVFYLLQAAIHGSEHVFCALAR | 622 | 646 | |  |  |
|  |  | LVVQCITSYWNNTR | 647 | 660 | |  |  |
|  |  | DLVAHVEALAQLVDDFTLTGPELGGQAQAELNHLMR | 692 | 727 | |  |  |
|  |  | DPALLPPLVWDCDALMR | 728 | 744 | |  |  |
|  |  | VSAGGHDPVYAAACNVATADFNR | 756 | 778 | |  |  |
|  |  | NDGQLLHNTQAR | 779 | 790 | |  |  |
|  |  | IYYYVMVPAFSR | 811 | 822 | |  |  |
|  |  | VVVDGPAMLTLQVLAHNMAER | 884 | 904 | |  |  |
|  |  | QVPLVPPALGANYFSSIR | 986 | 1003 | |  |  |
|  |  | ESAAGENALTYALMAGYFK | 1012 | 1030 | |  |  |
|  |  | TGLHPGFGFTVVR | 1042 | 1054 | |  |  |
|  |  | ASEAYFLGQLQVAR | 1069 | 1082 | |  |  |
|  |  | HETGGGVNFTLTQPR | 1083 | 1097 | |  |  |
|  |  | NPVTDMGNLPQNFYLGR | 1117 | 1133 | |  |  |
|  |  | GAPPLLDNAAAVYLR | 1134 | 1148 | |  |  |
|  |  | LGPAQPVPVFGCAQVPR | 1157 | 1173 | |  |  |
|  |  | AGMDHGQDAVCEFIATPVSTDVNYFR | 1175 | 1200 | |  |  |
|  |  | AFAATANPWASQR | 1237 | 1249 | |  |  |
|  |  | FSYGDLLYNGAYHLNGASPVLSPCFK | 1250 | 1275 | |  |  |
|  |  | ELVEDPCGLFQEAYPLTCASDPALLR | 1319 | 1344 | |  |  |
|  |  | SARNGEAHARETHFAQYLVYDASPLK | 1345 | 1370 | |  |  |
|  |  | NGEAHARETHFAQYLVYDASPLKGLAL | 1348 | 1374 | |  |  |
|  |  | ETHFAQYLVYDASPLKGLAL | 1355 | 1374 | |  |  |
| **VP26** | P10219/  Q25BW6 | GLVLATNNSQFIMDNNHPHPQGTQGAVR | 24 | 51 | | 36 | 33 |
|  |  | RTYSPFVVR | 96 | 104 | |  |  |
|  |  | TYSPFVVR | 97 | 104 | |  |  |
| **VP19c** | P32888/  B9VQG6 | TNPLPATPSVWGGSTVELPPTTR | 3 | 25 | | 257 | 55 |
|  |  | VLRPPISR | 36 | 43 | |  |  |
|  |  | AASTLWLLGLDGTDAPPGALTPNDDTEQALDK | 58 | 89 | |  |  |
|  |  | AASTLWLLGLDGTDAPPGALTPNDDTEQALDKILR | 58 | 92 | |  |  |
|  |  | QVILTDLCQPNADR | 112 | 125 | |  |  |
|  |  | AGTLLLALR | 126 | 134 | |  |  |
|  |  | HPADLPHLAHQR | 135 | 146 | |  |  |
|  |  | LGEAWGQLMEATALGSGR | 156 | 173 | |  |  |
|  |  | AGLVSFNFLVAACAASYDAR | 181 | 200 | |  |  |
|  |  | AHVTANYR | 208 | 215 | |  |  |
|  |  | FSECLR | 226 | 231 | |  |  |
|  |  | AMVHTHVFPHEVMR | 232 | 245 | |  |  |
|  |  | ITNTIHGTEDMTPPAPNR | 351 | 368 | |  |  |
|  |  | NPDFPLAGLAANPQTPR | 369 | 385 | |  |  |
|  |  | CSAGQVTNPQFADR | 386 | 399 | |  |  |
|  |  | TCTYAAFAELGMMPEDSPR | 415 | 433 | |  |  |
|  |  | FGAVSVPVVILEGVVWRPGEWR | 441 | 462 | |  |  |
| **VP23** | P10202/  B9VQE5 | MLADGFETDIAIPSGISRPDAAALQR | 1 | 26 | | 329 | 69 |
|  |  | VVFLPTIR | 31 | 38 | |  |  |
|  |  | QLTLADVAHESFVSGGVSPDTLGLLLAYR | 40 | 68 | |  |  |
|  |  | RRFPAVITR | 69 | 77 | |  |  |
|  |  | RFPAVITR | 70 | 77 | |  |  |
|  |  | IVACPLDVGLTHAGTVNLR | 83 | 101 | |  |  |
|  |  | NTSPVDLCNGDPISLVPPVFEGQATDVR | 102 | 129 | |  |  |
|  |  | LDSLDLTLR | 130 | 138 | |  |  |
|  |  | FPVPLPSPLAR | 139 | 149 | |  |  |
|  |  | NPGGLPDLNVLYYNGSR | 169 | 185 | |  |  |
|  |  | LSLLADVQQLGPVNAELR | 186 | 203 | |  |  |
|  |  | SLVLNMVYSITEGTTIILTLIPR | 204 | 226 | |  |  |
|  |  | LFALSAQDGYVNALLQMQSVTR | 227 | 248 | |  |  |
| **VP24** | P10210/  B9VQF3 | AVPIYVAGFLALYDSGDSGELALDPDTVR | 18 | 46 | | 279 | 38 |
|  |  | AALPPDNPLPINVDHR | 47 | 62 | |  |  |
|  |  | VLAVVDDPRGPFFVGLIACVQLER | 70 | 93 | |  |  |
|  |  | VLETAASAAIFER | 94 | 106 | |  |  |
|  |  | LLYLITNYLPSVSLATK | 117 | 133 | |  |  |
|  |  | LGGEAHPDR | 135 | 143 | |  |  |
|  |  | TLFAHVALCAIGR | 144 | 156 | |  |  |
|  |  | LGTIVTYDTGLDAAIAPFR | 158 | 176 | |  |  |
|  |  | LGTIVTYDTGLDAAIAPFRHLSPASR | 158 | 183 | |  |  |
|  |  | LAAEAELALSGR | 189 | 200 | |  |  |
|  |  | WSLVAER | 227 | 233 | |  |  |
|  |  | QAGIAGHTYLQASEKFK | 236 | 252 | |  |  |
|  |  | GYKNGAPESTDIPPGSIAAAPQGDRCPIVR | 265 | 294 | |  |  |
|  |  | RAARHSPGTNETITALMGAVTSLQQELAHMR | 466 | 496 | |  |  |
| **pUL17** | P10201/  B9VQE4 | GRPGLGTAANFQVEIQTR | 46 | 63 | | 97 | 41 |
|  |  | ASSAGGLFVSLPVVCDAQGVYDPYAVAALR | 104 | 133 | |  |  |
|  |  | VILFSYDELVPPNTR | 145 | 159 | |  |  |
|  |  | QGALVHQAVSGGAMGAADADAVLAGLEPPGGGR | 294 | 326 | |  |  |
|  |  | SLVEWLDRGWEALAGGDRPDWLWSR | 356 | 380 | |  |  |
|  |  | GWEALAGGDRPDWLWSR | 364 | 380 | |  |  |
|  |  | FVVVSYENSVAWGGR | 397 | 411 | |  |  |
|  |  | ARPPLLSSALATALTEACAAER | 413 | 434 | |  |  |
|  |  | VVRPHQLSPAGQAELLLR | 435 | 452 | |  |  |
|  |  | FPALEVPLR | 453 | 461 | |  |  |
|  |  | HPRPVLPPFDIAAEVAFTAR | 462 | 481 | |  |  |
|  |  | HPRPVLPPFDIAAEVAFTARIHLACLR | 462 | 488 | |  |  |
|  |  | IHLACLR | 482 | 488 | |  |  |
|  |  | RFPILLENLMR | 526 | 536 | |  |  |
|  |  | AVEGTAPDAFFHTAYALAVLAHLGGR | 537 | 562 | |  |  |
|  |  | FADSDGHYVFDYYSTSGDTLR | 580 | 600 | |  |  |
|  |  | VCEQYLPGESYAYLCLGFNR | 636 | 655 | |  |  |
| **pUL25** | P10209/  B9VQF2 | NFITPEFPR | 25 | 33 | 72 | | 41 |
|  |  | ETAAEQVVVLQAQR | 46 | 59 |  |  |  |
|  |  | YDTNLPVDLLHMVYAGR | 149 | 165 |  |  |  |
|  |  | TFMTALVLSLQACGRLYVGQR | 207 | 227 |  |  |  |
|  |  | NTHGAADDSDRAPVTFGDLLGRLPR | 245 | 269 |  |  |  |
|  |  | APVTFGDLLGRLPR | 256 | 269 |  |  |  |
|  |  | YLACLAAVIGTEGGRPQYR | 270 | 288 |  |  |  |
|  |  | YEHGALASHIVIATLMHHGVLPAAPGDVPR | 306 | 335 |  |  |  |
|  |  | GHNLFLWEDQTLLR | 363 | 376 |  |  |  |
|  |  | GHNLFLWEDQTLLRATANTITALGVIQR | 363 | 390 |  |  |  |
|  |  | LQLGMLIPGAVPSEAIAR | 406 | 423 |  |  |  |
|  |  | VVDMSSGARQAALVR | 486 | 500 |  |  |  |
|  |  | LTALELINRTR | 501 | 511 |  |  |  |
|  |  | TNPTPVGEVIHAHDALAIQYEQGLGLLAQQAR | 512 | 543 |  |  |  |
| **pUL36** | P10220 | SSLSFLSLIFDVGPR | 68 | 82 | 170 | | 27 |
|  |  | DVLSAEAIEGCLVEGGEWTR | 83 | 102 |  |  |  |
|  |  | MCSIVELPNFLEYPGAR | 112 | 128 |  |  |  |
|  |  | DGDTAYLFDPHGLPEGTPAFIAK | 189 | 211 |  |  |  |
|  |  | VRAGDMYPYLTYYTR | 212 | 226 |  |  |  |
|  |  | SPYPASPGLLELCVIFFFER | 570 | 589 |  |  |  |
|  |  | TAVGDFLASTRLSLADVAAHLPLVQHVLDENSLIGR | 626 | 661 |  |  |  |
|  |  | LSLADVAAHLPLVQHVLDENSLIGR | 637 | 661 |  |  |  |
|  |  | ETDAFYGELADLELQLR | 677 | 693 |  |  |  |
|  |  | SQAHPDTLFAPATPTHPEPLLYR | 712 | 734 |  |  |  |
|  |  | GVDAVSQHAGPLGVMPAPAGAAPQGAPRPPPLGPEAVQVR | 766 | 805 |  |  |  |
|  |  | FHVASAAVVPVVQLLESLPVFDQHTR | 846 | 871 |  |  |  |
|  |  | GQTLDAPEDLAAWLSVLTDAANQGLIER | 901 | 928 |  |  |  |
|  |  | FDALDAALGQQLDSDAAFVPAPGASPYPDDGGLSPEATR | 958 | 996 |  |  |  |
|  |  | LQGVLRPLPDFVGLK | 1057 | 1071 |  |  |  |
|  |  | ADMWGLLGQYR | 1108 | 1118 |  |  |  |
|  |  | FGGLLHAEGTAGDHSPSGR | 1299 | 1317 |  |  |  |
|  |  | ADELEAATADLREKMAAQR | 1333 | 1351 |  |  |  |
|  |  | AVTLALETALAFNPYTPENQR | 1412 | 1432 |  |  |  |
|  |  | IDWSAAFGAAADTYADMFR | 1445 | 1463 |  |  |  |
|  |  | LAGGLLERAQANDGFIDYHEAVLHLSEDLGGVPALR | 1475 | 1510 |  |  |  |
|  |  | AQANDGFIDYHEAVLHLSEDLGGVPALR | 1483 | 1510 |  |  |  |
|  |  | AIGSVALDLAAAAEEISAVR | 1540 | 1559 |  |  |  |
|  |  | SAEEIADQVEILVDQTEKAR | 1683 | 1702 |  |  |  |
|  |  | ELDVQAVAWLEHAQR | 1703 | 1717 |  |  |  |
|  |  | AACEQLRALQDTTNTVSGLRAQR | 1789 | 1811 |  |  |  |
|  |  | AGAVEELGGRVAQHADLSAR | 1832 | 1851 |  |  |  |
|  |  | ATGQTGAGAAAAPAPLLVDLR | 1911 | 1931 |  |  |  |
|  |  | ATGQTGAGAAAAPAPLLVDLRALDARAR | 1911 | 1938 |  |  |  |
|  |  | VSGGPGPLVLR | 1962 | 1972 |  |  |  |
|  |  | EATSTLDRPFAPSFLVPDGTPLQYALCFPAVTDK | 1973 | 2006 |  |  |  |
|  |  | LQLALSDAQAANFQLFGR | 2046 | 2063 |  |  |  |
|  |  | HRQARWGASMDAAAELYVALVATTLTR | 2067 | 2093 |  |  |  |
|  |  | WGASMDAAAELYVALVATTLTR | 2072 | 2093 |  |  |  |
|  |  | WAQLEWGGDAAAPGPPLGPQSSTR | 2099 | 2122 |  |  |  |
|  |  | VSFNENDVLVALVASSPEHIYTFWR | 2125 | 2149 |  |  |  |
|  |  | QHEYMHLTLPRAFQNAADSMLFVQRLTPHPDAR | 2155 | 2187 |  |  |  |
|  |  | GKLSETDPLAPWRSVPELGTER | 2216 | 2237 |  |  |  |
|  |  | ESELELCLTLFDSRARGPDAALR | 2367 | 2389 |  |  |  |
|  |  | GPPCLVLVDISMTPVAVLWENPDPPGPPDVR | 2435 | 2465 |  |  |  |
|  |  | FVGSEATEELPFVAGGEDVLAASATDEDPFLAR | 2466 | 2498 |  |  |  |
|  |  | AILGRPFDASLLSGELFPGHPVYQR | 2499 | 2523 |  |  |  |
|  |  | ALLHHSDAVLTSLHHVRMLLG | 3144 | 3164 |  |  |  |
| **N-pUL36** | P1022 | SSLSFLSLIFDVGPRDVLSAEAIEGCLVEGGEWTR | 68 | 102 | 118 | | 20* |
|  |  | LSLADVAAHLPLVQHVLDENSLIGR | 637 | 661 |  |  |  |
|  |  | ETDAFYGELADLELQLR | 677 | 693 |  |  |  |
|  |  | SQAHPDTLFAPATPTHPEPLLYR | 712 | 734 |  |  |  |
|  |  | FGGLLHAEGTAGDHSPSGR | 1299 | 1317 |  |  |  |
|  |  | AQANDGFIDYHEAVLHLSEDLGGVPALR | 1483 | 1510 |  |  |  |
|  |  | AIGSVALDLAAAAEEISAVR | 1540 | 1559 |  |  |  |
|  |  | ATGQTGAGAAAAPAPLLVDLR | 1911 | 1931 |  |  |  |
|  |  | LQLALSDAQAANFQLFGR | 2046 | 2063 |  |  |  |
|  |  | WGASMDAAAELYVALVATTLTR | 2072 | 2093 |  |  |  |
|  |  | VSFNENDVLVALVASSPEHIYTFWR | 2125 | 2149 |  |  |  |
|  |  | GPPCLVLVDISMTPVAVLWENPDPPGPPDVR | 2435 | 2465 |  |  |  |
|  |  | FVGSEATEELPFVAGGEDVLAASATDEDPFLAR | 2466 | 2498 |  |  |  |
| **pUL37** | P10221/  B9VQG5 | GLPSEAPVVTTSPAGPPSDGPMQR | 5 | 28 | 128 | | 36 |
|  |  | AHGLPETALLAENLPGLLVHR | 97 | 117 |  |  |  |
|  |  | LVVALPEAPDQAFR | 118 | 131 |  |  |  |
|  |  | TAAALGPVRVR | 159 | 169 |  |  |  |
|  |  | SCLAMSPRTSIEALGETSLK | 185 | 204 |  |  |  |
|  |  | MAPVPLGQPSANLTTPAYSLLFPAPFVQEGLR | 205 | 236 |  |  |  |
|  |  | ALFTLALVDEYLTTPER | 266 | 282 |  |  |  |
|  |  | GAVVPPPLLAQFQHTVR | 283 | 299 |  |  |  |
|  |  | DFGLGYLPTVEGHR | 658 | 671 |  |  |  |
|  |  | GGLGPTMQMADNIEQLLR | 687 | 704 |  |  |  |
|  |  | GGLGPTMQMADNIEQLLRELYVISR | 687 | 711 |  |  |  |
|  |  | GAVEQLRPLVQLQPPPPPEVGTSLLLISMYALAAR | 712 | 746 |  |  |  |
|  |  | LGPWPPEAMGDAVSQYCSMYHDAK | 803 | 826 |  |  |  |
|  |  | SVITETTAHLGVCDELAAQVSHEDNVLAVVR | 838 | 868 |  |  |  |
|  |  | EIHGFLSVVSGIHAR | 870 | 884 |  |  |  |
|  |  | LLSGDQVPGFCFMGQFLAR | 888 | 906 |  |  |  |
|  |  | AAAGPEPVAEFVQELHDTWK | 919 | 938 |  |  |  |
|  |  | RDLGAWGDYSLGPLGQTTAVPDSVDLSR | 989 | 1016 |  |  |  |
|  |  | DLGAWGDYSLGPLGQTTAVPDSVDLSR | 990 | 1016 |  |  |  |
|  |  | ASAFRPLAGPESPR | 1043 | 1056 |  |  |  |
|  |  | DAGNSLPAPMPMDAQKPEAYGHGPR | 1062 | 1086 |  |  |  |
| **pUL16** | P10200/  B9VQE3 | RPLAPPGPPGTLPRPDSR | 8 | 25 | 53 | | 49 |
|  |  | DRVDDLGTDVDSIAR | 33 | 47 |  |  |  |
|  |  | IVNSVFVWR | 48 | 56 |  |  |  |
|  |  | CLTVLTEPLCQVALPNPDPGR | 69 | 89 |  |  |  |
|  |  | ALFCEIFLYLTRPK | 90 | 103 |  |  |  |
|  |  | LPPNTFFALFFFNR | 107 | 120 |  |  |  |
|  |  | SVTHPLTPLLCTLTFAR | 132 | 148 |  |  |  |
|  |  | AATPPEETPDPTTEQLAEEPVVGELDGAYLVPAK | 151 | 184 |  |  |  |
|  |  | HLGWLLAR | 229 | 236 |  |  |  |
|  |  | MAQCTLAVQGDASLCPLLFGHPVDTVTLLQAPR | 280 | 312 |  |  |  |
| **pUL21** | P10205 | AYFVCGGCVYSVGRPCASQPGEIAK | 25 | 49 | 83 | | 31 |
|  |  | IIELFEHPTIVNVSSHFVYTPSPYVFALAQAHLPR | 145 | 179 |  |  |  |
|  |  | LPSSLEALVSGLFDGIPAPR | 180 | 199 |  |  |  |
|  |  | VGPAGVSPAPPPNNTDSSSLVPGAQDSAPPGPTLR | 251 | 285 |  |  |  |
|  |  | ELWWVFYAADR | 286 | 296 |  |  |  |
|  |  | ALEEPRADSGLTR | 297 | 309 |  |  |  |
|  |  | LAVPGGVISPEHVAYLGAFLSVLYAGR | 444 | 470 |  |  |  |
| **VP13/14** | P10231 | RASTRPRASPVADEPAGDGVGFMGYLR | 12 | 38 | 308 | | 58 |
|  |  | ASTRPRASPVADEPAGDGVGFMGYLR | 13 | 38 |  |  |  |
|  |  | ASPVADEPAGDGVGFMGYLR | 19 | 38 |  |  |  |
|  |  | GDDDSELEALEEMAGDEPPVR | 43 | 63 |  |  |  |
|  |  | QGGYLGPVDAR | 121 | 131 |  |  |  |
|  |  | VPGPELR | 184 | 190 |  |  |  |
|  |  | LAPQVAVWDESVR | 198 | 210 |  |  |  |
|  |  | SALALGHPAGFYPCPDSAFGLSR | 211 | 233 |  |  |  |
|  |  | VGVMHFASPDNPAVFFR | 234 | 250 |  |  |  |
|  |  | QTLQQGEALAWYITGDGILDLTDR | 251 | 274 |  |  |  |
|  |  | QTLQQGEALAWYITGDGILDLTDRR | 251 | 275 |  |  |  |
|  |  | TSPAQAMSFLADAVVR | 278 | 293 |  |  |  |
|  |  | LAINGWVCGTR | 294 | 304 |  |  |  |
|  |  | QFASLTALRPVGAAAVPLLSAGGLVSPQSGPDAAVFR | 324 | 360 |  |  |  |
|  |  | SSLGSLLYWPGVR | 361 | 373 |  |  |  |
|  |  | MTYLATGALLAR | 390 | 401 |  |  |  |
|  |  | VLDVLAVMAEQTVQWLSVVVGAR | 421 | 443 |  |  |  |
|  |  | LHPHVHHPAFADVAR | 444 | 458 |  |  |  |
|  |  | ALPLGSPAVVGAEHEALGDTAAR | 464 | 486 |  |  |  |
|  |  | LLANSGLNAVLGAAVYALHTALATVTLK | 488 | 515 |  |  |  |
|  |  | AILAAGLVLQR | 535 | 545 |  |  |  |
|  |  | LLGFADTVVACVTLAAFDGGFTAPEVGTYTPLR | 546 | 578 |  |  |  |
|  |  | FWADVR | 598 | 603 |  |  |  |
|  |  | DLEPFPPAPVSGGSVLGPR | 634 | 652 |  |  |  |
|  |  | VVDIMSQFR | 655 | 663 |  |  |  |
| **VP16** | P06492 | NTPAAPPLYATGR | 30 | 42 | 264 | | 51 |
|  |  | LSQAQLMPSPPMPVPPAALFNR | 43 | 64 |  |  |  |
|  |  | FLSTLPSDVVEWGDAYVPER | 104 | 123 |  |  |  |
|  |  | AHGDVAFPTLPATR | 130 | 143 |  |  |  |
|  |  | DGLGLYYEALSR | 144 | 155 |  |  |  |
|  |  | FFHAELR | 156 | 162 |  |  |  |
|  |  | TVLANFCSALYR | 170 | 181 |  |  |  |
|  |  | DRDLGEMLR | 200 | 208 |  |  |  |
|  |  | VLFLHLYLFLTR | 225 | 236 |  |  |  |
|  |  | QLAGLFQPFMFVNGALTVR | 265 | 283 |  |  |  |
|  |  | GVPIEAR | 284 | 290 |  |  |  |
|  |  | EHLNLPLVR | 300 | 308 |  |  |  |
|  |  | SAATEEPGAPLTTPPTLHGNQAR | 309 | 331 |  |  |  |
|  |  | ASGYFMVLIR | 332 | 341 |  |  |  |
|  |  | LDSYSSFTTSPSEAVMR | 344 | 360 |  |  |  |
|  |  | EHAYSR | 361 | 366 |  |  |  |
|  |  | NNYGSTIEGLLDLPDDDAPEEAGLAAPR | 371 | 398 |  |  |  |
|  |  | LSFLPAGHTR | 399 | 408 |  |  |  |
| **VP22** | P10233 | RGALQTRSR | 42 | 50 | 313 | | 60 |
|  |  | FVQYDESDYALYGGSSSEDDEHPEVPR | 57 | 83 |  |  |  |
|  |  | RPVSGAVLSGPGPAR | 86 | 100 |  |  |  |
|  |  | APRTQR | 121 | 126 |  |  |  |
|  |  | VATKAPAAPAAETTRGR | 127 | 143 |  |  |  |
|  |  | SAQPESAALPDAPASTAPTR | 145 | 164 |  |  |  |
|  |  | LHFSTAPPNPDAPWTPR | 176 | 192 |  |  |  |
|  |  | VAGFNKRVFCAAVGR | 193 | 207 |  |  |  |
|  |  | TDEDLNELLGITTIR | 228 | 242 |  |  |  |
|  |  | ANELVNPDVVQDVDAATATR | 255 | 274 |  |  |  |
|  |  | SAASRPTERPRAPARSASRPR | 277 | 297 |  |  |  |
| **VP11/12** | P10230 | LARCLTPANLIR | 13 | 24 | 295 | | 40 |
|  |  | IFGGCLLPTPEGLLSAAVGALR | 35 | 56 |  |  |  |
|  |  | QRSDDAQPAFLTCTDRSVR | 57 | 75 |  |  |  |
|  |  | SDDAQPAFLTCTDRSVRLAAR | 59 | 79 |  |  |  |
|  |  | QHNTVPESLIVDGLASDPHYEYIR | 80 | 103 |  |  |  |
|  |  | YLQTVVPSGLDVPEDPVGDCDPSLHVLLRPTLAPK | 134 | 168 |  |  |  |
|  |  | LNELLAYVSVLYR | 221 | 233 |  |  |  |
|  |  | WASWMLWTTDK | 234 | 244 |  |  |  |
|  |  | FLPLGGSPEAPAETFAR | 257 | 273 |  |  |  |
|  |  | AAVSDVLGHLTRLANLWQTGK | 293 | 313 |  |  |  |
|  |  | TTAPLFPTMTAPSWAR | 376 | 391 |  |  |  |
|  |  | AWFGAALAADLLR | 398 | 410 |  |  |  |
|  |  | AWFGAALAADLLRNGAPSLHYESILR | 398 | 423 |  |  |  |
|  |  | DNEPPPLPRPRLHSTPASTR | 465 | 484 |  |  |  |
|  |  | HAPYEDDESIYETVSEDGGR | 603 | 622 |  |  |  |
|  |  | VYEEIPWMR | 623 | 631 |  |  |  |
|  |  | TGPPPPPLSPSPVLAR | 671 | 686 |  |  |  |

The protein composition of HSV1 capsids, virions sedimented from cell culture supernatants and gradient purified virions were analyzed by quantitative mass spectrometry. Under the experimental conditions, Mowse scores exceeding 33 are significant at the 95% level. Scores and sequence coverages of the experiment with the highest score are given.
*Calculated on basis of the full length pUL36.
