# Supplementary figures and images for: Plus- and Minus-End Directed Microtubule Motors Bind Simultaneously to Herpes Simplex Virus Capsids Using Different Inner Tegument Structures
Source: PLoS Pathog. 2010 Jul 8;6(7):e1000991. doi: 10.1371/journal.ppat.1000991 (PMC2900298; doi:10.1371/journal.ppat.1000991)

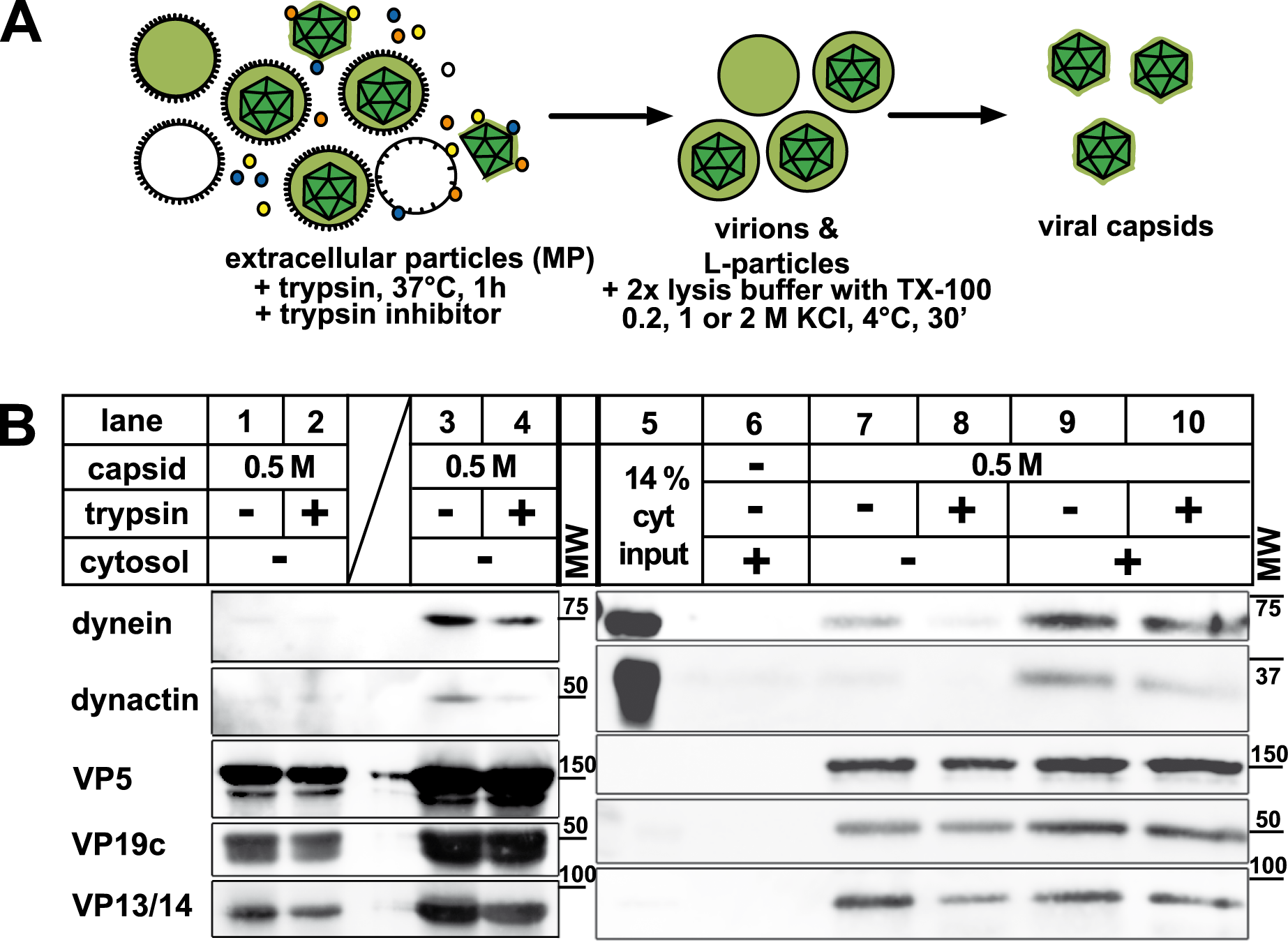

Supplement: Figure S1 — Preparation of HSV1 capsids lacking host proteins. A. Extracellular particle preparations contain virions (dark+light green), light particles consisting of a membrane and tegument but no capsid (light green), unenveloped and/or broken capsids (dark green) with associated motors and other host cell proteins (small, colored circles). The extracellular particles sedimented from the medium of infected cells (MP, medium pellet) were therefore incubated with trypsin for 1 h, and subsequently with an excess of trypsin inhibitor to remove pre-bound host factors while protecting viral proteins by the viral envelope. Such pretreated particles were then lysed with a buffer containing TX-100 and different amounts of KCl to isolate viral capsids by sedimentation through sucrose cushions (c. f. Fig. 1). B. Immunoblot analysis of viral particles untreated or incubated with trypsin and of their ability to recruit MAPs. Extracellular particles of HSV1(F) corresponding to 5×10^8^ pfu were either left untreated in buffer (lanes 1, 3, 7, 9) or incubated with trypsin (lanes 2, 4, 8, 10) prior to addition of trypsin inhibitor and lysis with TX-100 in the presence of 0.5 M KCl. Viral capsids were sedimented, incubated in buffer (lanes 1 to 4, 7, 8) or 0.75 mg/ml pig brain cytosol (lanes 9, 10), and sedimented through sucrose cushions. The resulting pellets were analyzed by immunoblot using antibodies against against dynein (lanes 1 to 4, MAB1618 against intermediate chain; lanes 5 to 10, anti-LIC2 against light intermediate chain), and dynactin (lanes 1 to 4, mAb anti-p50; lanes 5 to 10, mAb3F2.3 against CapZβ). The amount of capsids in each sample was estimated by labeling the major capsid proteins VP5 (pAb NC-1) or VP19c (pAb NC-2) or the outer tegument protein VP13/14 P26 (pAb R220). As controls, the cytosol was also directly analyzed (lane 5), or sedimented in the absence of capsids (lane 6). Please note that the two different HSV1(F) preparations (lanes 1 and 2 versus lanes 3 and 4) co [file ppat.1000991.s005.tif]

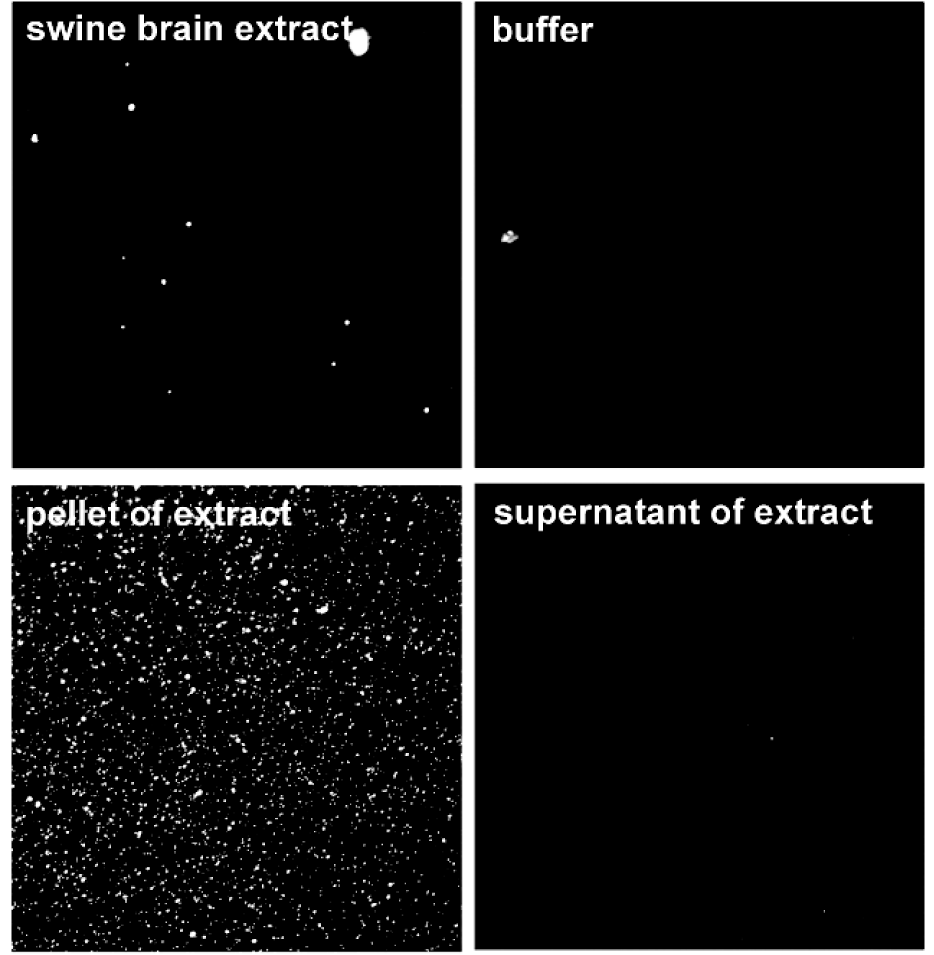

Supplement: Figure S2 — The mammalian cytosol fraction does not contain membranes. Pig brain extract or buffer were incubated with 1 µM DiIC18 (Molecular Probes, Eugene, USA) for 30 min at room temperature and subsequently centrifuged as described before (Wolfstein et al. 2006). Labeled membranes in the brain extract, buffer as a control, the pellet and the supernatant of the centrifugation were analyzed by fluorescence microscopy. Only traces of membranes were detected in the cytosolic supernatant. (0.18 MB TIF) [file ppat.1000991.s006.tif]
